# Microbial biotransformation of *Syzygium australe* modifies metabolomic profile assessed with multivariate analysis and molecular networking: In vitro and computational studies

**DOI:** 10.1038/s41598-026-55423-x

**Published:** 2026-06-14

**Authors:** Heba H. Elzayat, Noha A. Seif -Eldein, Lina J. M. Abdel-Hafez, Hussein N. Ghanem, Alaadin E. El-Haddad, Shaza A. Mohamed

**Affiliations:** 1 Pharmacognosy Department, Faculty of Pharmacy, October 6 University, Giza, Egypt; 2 Pharmacognosy and Medicinal Plants Department, Faculty of Pharmacy (Girls), Al-Azhar University, Nasr City, 11754 Cairo Egypt; 3 Microbiology and Immunology Department, Faculty of Pharmacy, October University, Giza, Egypt; 4 Chemistry Department, Faculty of Pharmacy, October 6 University, October City, Giza, 12585 Egypt

**Keywords:** LC-MS/MS, Sulfated flavonoids, GNPS, Docking, α-glucosidase, α-amylase, Pancreatic lipase, Antioxidant, Biochemistry, Biological techniques, Biotechnology, Drug discovery, Microbiology

## Abstract

*Syzygium australe*, a comparatively less studied species within the *Syzygium* genus, is emerging as a prospective source of bioactive phytochemicals. In this study, the impact of microbial biotransformation by *Aspergillus niger* on the metabolomic and bioactivity profiles of *S. australe* leaves extract (SAE) was evaluated. UPLC-T-TOF-MS/MS and molecular networking enabled the tentative identification of 80 metabolites in SAE, with flavonoids emerging as the dominant phytoconstituents. After biotransformation, sulfated flavonoids are the main metabolites in *S. australe* biotransformed extract (SABE), suggesting that enzymatic sulfonation is mediated by fungal sulfotransferase enzymes. Molecular networking revealed two key clusters: cluster A, which is primarily composed of quercetin derivatives, and cluster B, which corresponded to syringetin. Notably, the biotransformed metabolites in SABE were predominantly observed as self-looped nodes, indicating the formation of structurally unique compounds. Multivariate chemometric analyses revealed a significant metabolomic modulation and a clear discrimination between SAE and SABE. Compared with SABE, SAE significantly increased the free radical scavenging capacity, as evidenced by lower IC₅₀ values in DPPH and ABTS assays (36.96 ± 1.20 and 19.80 ± 0.85 *µ*g/mL respectively), which is likely a consequence of tannin degradation during microbial biotransformation. The bioactivity of SABE, particularly against pancreatic lipase, was enhanced, with an inhibition rate of 74.49 ± 4.80% at 100 *µ*g/mL. Molecular docking further supported these findings, highlighting isorhamnetin-3-*O*-sulfate as a key bioactive constituent with the highest binding affinity to pancreatic lipase (ΔG = − 12.47 kcal/mol). These findings highlight a significant potential and warrant further investigation using alternative microbial strains aiming to develop novel therapeutic agents.

**Supplementary Information:**

The online version contains supplementary material available at 10.1038/s41598-026-55423-x.

## Introduction

The Myrtaceae family has garnered increasing interest because of its rich diversity and ethnopharmacological importance. Over 5,650 species and more than 130 genera were identified^1^. Myrtaceae includes genera such as *Eucalyptus*, *Psidium*, *Feijoa*, and *Syzygium*, which are traditionally used for treating metabolic and infectious diseases. *Syzygium* has demonstrated significant antidiabetic, antiobesity, and antioxidant properties^2^. For example, *S. alternifolium* and *S*. *aqueum* extracts significantly reduce blood glucose levels in diabetic rats^3,4^. *Syzygium australe*, commonly known as brush cherry or Australian cherry, is native to the eastern coast of Australia and is a relatively underexplored member of its genus. Although it is traditionally cultivated for ornamental purposes, its phytochemical profile and potential medicinal properties are gaining attention^5^.

Targeted inhibition of essential digestive enzymes, especially α-amylase and α-glucosidase are widely recommended treatments for patients with type 2 diabetes mellitus^6^. Pancreatic lipase inhibitors are used to reduce fat absorption and protect pancreatic integrity^7^. Controlling oxidative stress can lower the risk of diabetes and its cardiovascular effects^8^. In this context, polyphenol-rich plants and foods have emerged as promising agents because of their dual role in enzyme inhibition and antioxidant activity^9^.

Microbial biotransformation has emerged as a cutting-edge strategy to modify chemical structures, often yielding derivatives with improved solubility, bioavailability, or biological activity^10^. *Aspergillus niger* (*A. niger*) is one of the most versatile and industrially valuable species. *A. niger* has been extensively used in the biotransformation of flavonoids, terpenoids, alkaloids, and phenolics^11^, because its enzymatic arsenal is capable of catalyzing diverse reactions, including oxidation, hydrolysis, isomerization, and functional group modifications^12^. Metabolomics represents a powerful systems-level approach designed for the untargeted, high-throughput exploration of complex metabolite compositions. UPLC-MS/MS and molecular networking offer sophisticated means to organize and visualize MS/MS fragmentation data on the basis of spectral similarity. This facilitates rapid dereplication and the discovery of novel metabolites^13^. The integration of such analytical pipelines with advanced multivariate statistical tools, including principal component analysis (PCA), hierarchical clustering analysis (HCA), partial least squares–discriminant analysis (PLS-DA), orthogonal PLS-DA (OPLS-DA), and volcano plot analysis, provides a powerful framework for comprehensive metabolomic data interpretation, enabling accurate sample discrimination and proper identification of key discriminatory biomarkers^14^.

This research aims to explore the impact of *A. niger* biotransformation on *S. australe* leaves extract, followed by comprehensive untargeted metabolomic profiling via UPLC-T-TOF-MS/MS. The metabolites were visualized via GNPS-based molecular networking and analysed by multivariate statistical methods to identify distinct chemical features. Additionally, this study investigated the in vitro antioxidant activity and inhibitory potential against α-amylase, α-glucosidase, and lipase enzymes, alongside molecular docking verification. This study represents the first integrative approach combining fungal biotransformation with advanced metabolomics tools to systematically elucidate chemical alterations and correlate them with biological activities in *S. australe*. Furthermore, this work provides novel insights into the role of *A. niger*-mediated metabolic modulation in enhancing bioactive profiles, thereby offering a promising strategy for the discovery of value-added phytoconstituents with potential therapeutic applications.

## Materials and methods

### Reagents and enzymatic agents

All reagents were of high analytical grade. The enzymes α-amylase from pig pancreas (Catalogue No. A3176), α-glucosidase from *Saccharomyces cerevisiae* (Catalogue No. G5003), and pancreatic lipase from pig pancreas (Catalogue No. L3126) were purchased from Sigma‒Aldrich (St. Louis, MO, USA). Substrates, namely, 2-chloro-4-nitrophenyl-α-D-maltotrioside (Catalogue No. 93834), p-nitrophenyl β-D-glucopyranoside (Catalogue No. N7006), and p-nitrophenyl dodecanoate (Catalogue No. 61716), were also supplied by Sigma‒Aldrich (St. Louis, MO, USA). Reference standards used in the bioactivity assays included **Trolox** (Catalogue No. 238813), **acarbose** (Catalogue No. A8980), and **orlistat** (Catalogue No. O4139), all purchased from Sigma-Aldrich (St. Louis, MO, USA).

### Plant materials and extraction

Fresh mature leaves of *S. australe* J.C. Wendl. ex-Link were collected in January 2022 from a botanical garden at El Barageel, Giza, Egypt, with official permission granted by *Prof*. Dr. Treez Labib, Director of the botanical garden. The plant was identified by *Prof*. Dr. Abd-Elhalim Abd-Elmagli, a taxonomy specialist at the Agricultural Research Centre, Dokki, Egypt. A voucher specimen (No. SE 2022 − 265) was deposited at the herbarium of the Department of Pharmacognosy and Medicinal Plants, Faculty of Pharmacy (Girls), Al-Azhar University, Cairo, Egypt. Leaves were collected from 5 independent plants (biological replicates) and processed separately. Leaves were thoroughly washed with tap water, dried in an oven (40 °C) and then finely grounded. Each replicate powder (0.5 kg) was first defatted with *n*-hexane (3 × 3 L) at room temperature for 24 h each cycle with occasional stirring to remove lipophilic constituents. The defatted powdered materials were air-dried to remove residual solvent and subsequently macerated in ethanol (70%, 5 L) at room temperature (25 ± 2 °C) for 72 h with occasional stirring. The extract was filtered through Whatman No. 1 filter paper and the mixture was repeated twice under the same conditions to ensure exhaustive extraction. The combined filtrates were concentrated under reduced pressure via a rotary evaporator (Rotavapor^®^, BÜCHI, Switzerland) at 40 °C until a semisolid residue was obtained. All extracts were processed independently for downstream analyses^15^.

### Microbial biotransformation

*A. niger* (ATCC 10404), obtained from the Microbiological Resources Centre (MIRCEN) (Ain Shams University, Cairo, Egypt), was cultivated in Sabouraud dextrose broth (SDB) for 14 days at 25 ± 2 °C. A spore suspension (10⁷ CFU/mL in Phosphate-Buffered Saline (PBS) with 5 *µ*L/mL Tween-20) was prepared and activated by incubation (28 °C, 48 h, 180 rpm). A preliminary time-course study was conducted in which samples were collected after 14, 21, and 28 days, and the resulting metabolite profiles were comparatively analyzed using HPLC. The chromatographic results revealed that the cultures incubated for 14 days exhibited the highest metabolite diversity along with the greatest relative peak intensities. Accordingly, a 14-day incubation period was selected as the optimal condition for all subsequent large-scale experiments^16^. To ensure rigorous interpretation of the metabolomic changes from background signals, two independent control systems were processed under identical experimental conditions. A fungal control experiment consisting of *A. niger* cultured in SDB without SAE was included to identify metabolites originating from fungal metabolism. In parallel, a substrate control experiment consisting of SAE incubated in sterile SDB without fungal inoculation was used to assess non-enzymatic degradation and medium-related interactions. Representative HPLC chromatograms of the treated samples and controls are presented in Fig. S1. For the preparative biotransformation experiment, the activated spore suspension (5 mL) was inoculated into Erlenmeyer flasks (500 mL) containing SDB (200 mL). SAE (60 g) was initially suspended in a minimal volume of dimethyl sulfoxide (DMSO), then diluted with distilled water (20 mL) prior to addition to the culture medium, ensuring that the final DMSO concentration did not exceed 1% (v/v) a level widely reported as non-inhibitory to the microbial viability^17–19^. The cultures were incubated at 25 ± 2 °C under continuous shaking at 180 rpm for 14 days. The initial pH of the medium was approximately 5.6 and decreased to 4.3 ± 0.2 after fermentation, consistent with organic acid production by *A. niger*. All experiments were carried out in five independent biological replicates, each initiated from separately prepared spore suspensions and SAE to ensure reproducibility^20^.

### **UPLC-T-TOF-MS**/**MS analysis**

The analysis of SAE and SABE was conducted at the Proteomics and Metabolomics Unit, Children’s Cancer Hospital, Cairo, Egypt, using an Exion LC system coupled to a Triple TOF 5600 + mass spectrometer (SCIEX, Framingham, MA, USA). Chromatographic separation was carried out on an XSelect HSS T3 C18 column (Waters Corporation, CT, USA). The mobile phase consisted of solvent A (5 mM ammonium formate buffer, pH 8, containing 1% methanol) and solvent B (acetonitrile), and the separation was performed at a flow rate of 0.3 mL/min via the following gradient program: 0–1 min, 90% A and 10% B (isocratic); 1.1–20.9 min, a linear gradient to 10% A and 90% B; 21–25 min, 10% A and 90% B (isocratic); followed by reequilibration to initial conditions (90% A and 10% B) from 25.1 to 35 min. Sample preparation was carried out by extracting 50 mg of each sample in 1 mL of a mixture of deionized water, methanol, and acetonitrile (50:25:25,v/v/v), followed by vortex mixing for 2 min, ultrasonication for 10 min, and centrifugation at 10,000 rpm for 5 min. An aliquot of the supernatant (50 *µ*L) was diluted with 1000 mL of reconstitution solvent to obtain a final concentration of 2.5 *µ*g/mL, further 10 *µ*L was injected for analysis. To ensure analytical robustness and reproducibility, each analysis was performed in five technical replications. Mass spectrometric detection was performed in negative electrospray ionization (ESI^−^) mode, and data were acquired in both full-scan MS and data-dependent MS/MS modes to ensure comprehensive metabolite profiling and reliable structural characterization^21^.

### GNPS plot generation and data interpretation

A molecular network (MN) was created via UPLC-T-TOF-MS/MS data obtained from both SAE and SABE samples. The data were uploaded to the GNPS platform via WinSCP (https://winscp.net), where the MN was generated via the GNPS online procedure^22^. The creation of the network was guided by specific parameters, including a minimum cosine score of 0.65, a parent mass tolerance of 0.1 Da, and a fragment ion tolerance of 0.5 Da for generating consensus spectra. The network also requires at least four matched peaks and a minimum cluster size of 1. MS clustering and filtering options were activated. The resulting molecular network was subsequently analysed and visualized via Cytoscape (version 3.10.2 software)^14^.

### Multivariate data analysis

The raw LC-MS data were converted to mzML format via ProteoWizard (msConvert) and processed via MS-DIAL to generate a data matrix of *Rt*, *m/z*, and peak intensities. Low-quality features were removed, and missing values were imputed prior to analysis. The dataset was imported into MetaboAnalyst 6.0. A total of five replicates per group were included. Data were normalized by median normalization, followed by log10 transformation and autoscaling to reduce systematic variation and enhance comparability. Unsupervised PCA was performed to assess the data structure and detect outliers, with model performance evaluation. Supervised models (PLS-DA and OPLS-DA) were applied for group discrimination and validated via R²Y, Q²Y, permutation testing (*n* = 1000), and CV-ANOVA (*p* < 0.05). Discriminant metabolites were selected on the basis of variable importance in projection (VIP > 1.0) combined with correlation coefficients and visualized via **S-**plots and loading plots. HCA was performed via Euclidean distance and Ward’s linkage methods to further confirm clustering consistency. Additionally, a PLS biplot was generated to explore metabolite contributions to sample discrimination. Univariate analysis was conducted via a volcano plot, which integrates the fold change (log₂FC) and statistical significance (p value), to identify significantly altered metabolites^23^.

### ABTS and DPPH-based in vitro antioxidant screening

ABTS and DPPH tests were used to assess the antioxidant properties of SAE and SABE. The ABTS test was performed following **Arnao** et al. **(2001)**^24^ with modifications by **Elkholy** et al. **(2023)**^25^, using Trolox as a reference antioxidant. The DPPH test was carried out using Trolox as the reference standard and the procedure outlined by **Boly** et al. **(2016)**^26^, which was altered by **Elkholy** et al. **(2023)**^25^. The absorbance was determined with a microplate reader (Omega, USA). A negative control representing 100% radical activity was prepared by replacing the tested extracts with the corresponding solvent under identical experimental conditions. Additionally, sample blanks were included to correct for any intrinsic absorbance or potential interference from the extracts. The antioxidant activity for both assays was expressed as a percentage inhibition, which was calculated via the following formula: % inhibition = [(∆A _control_ – ∆A _sample_)/∆A _control_] × 100. GraphPad Prism 9 was used to analyse the data by fitting a nonlinear regression model (log[inhibitor] vs. normalized response, variable slope) to determine the IC₅₀ values.

### In vitro evaluation of the inhibition of α-glucosidase, α-amylase, and pancreatic lipase enzymes

The α-glucosidase inhibition test was conducted according to the protocol described by **Abdallah** et al. **(2022)**^27^, with acarbose used as a reference inhibitor. The absorbance of *p*-nitrophenol, which is liberated from the pNPG substrate, was measured at 405 nm via a microplate reader (Omega, USA) to track enzyme activity. Following the procedure outlined by **Khadayat** et al. **(2020)**^28^, the α-amylase inhibition assay was performed with acarbose as a positive control. The reaction was quantified by detecting the formation of *p*-nitroaniline at 405 nm.

The lipase inhibition assay was executed following the method of **Abdallah** et al. **(2022)**^27^, with orlistat as a reference standard. Lipase activity was assessed by measuring the absorbance of *p*-nitrophenol, a product of *p*-NPD substrate hydrolysis, at 405 nm via an Omega microplate reader. A negative control representing 100% enzyme activity was prepared by substituting the tested extracts with the corresponding solvent under identical experimental conditions. Additionally, sample blanks (extract controls in the absence of enzyme) were included to correct for any intrinsic absorbance or potential interference arising from the extracts. The inhibitory activity of each enzyme was calculated according to the following equation: % inhibition = [(∆A _control_ – ∆A _sample_)/∆A _control_] × 100.

### Molecular docking study

The Molecular Operating Environment (MOE) 2019.0102 was utilized for this study. The three-dimensional structure of the pancreatic lipase–colipase complex bound to a C11 alkyl phosphonate inhibitor (PDB ID: 1LPB) was obtained from the RCSB Protein Data Bank. The protein was simplified and protonated. Before docking, the receptor placement was adjusted to the alpha triangle and then refined using a rigid receptor, a scoring method based on the London DJ scoring function. Key amino acids involved in catalytic activity were identified on the basis of previous literature^29^. The target compounds were then compared with orlistat^30^.

## Results and discussion

### Metabolite profiling of SAE and SABE via UPLC-T-TOF-MS/MS

After extraction, SAE yielded 12–18% w/w across 5 independent biological replicates. The variation observed among replicates is primarily attributable to minor experimental variability during the extraction process. However, after biotransformation, the recovered yield was 75–97% w/w (45–58 g per 60 g of SAE), depending on the biological replicate and fermentation batch. This recovered mass represents a complex metabolite matrix comprising untransformed residual phytoconstituents together with newly generated fungal biotransformation products formed during the incubation period.

**Fig. 1:**
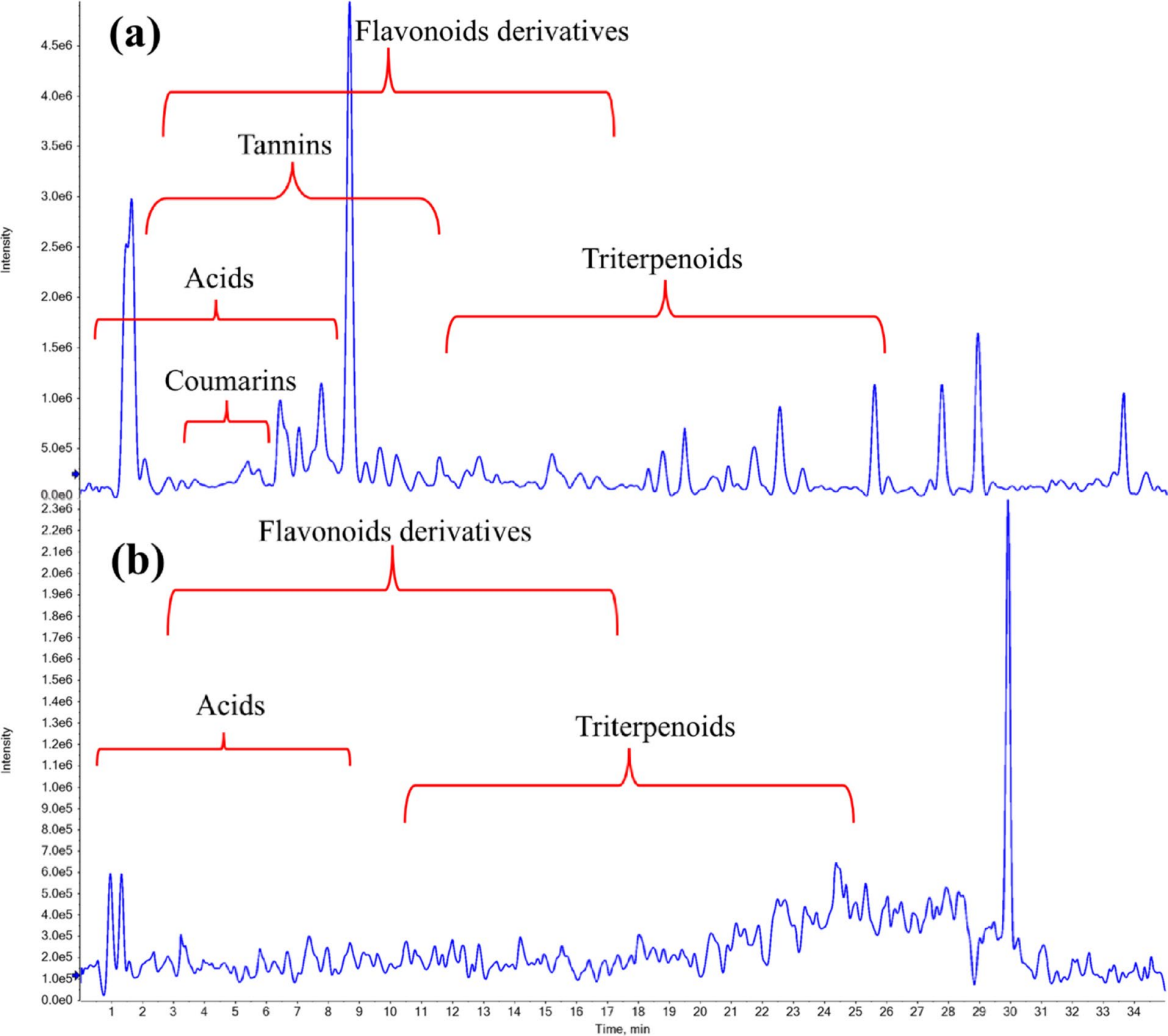
Base peak chromatograms of the UPLC-tandem mass spectrometry results of the **(a);** *S. australe* leaves extract (SAE) and **(b);** *S. australe* biotransformed extract (SABE) in negative ionization mode.

UPLC-MS/MS was used to analyse the metabolite profiles of SAE and SABE. The detection was performed in negative ionization mode. Representative chromatograms of both samples are depicted in Fig. 1. UPLC-MS led to the identification of 80 unique metabolites in SAE, reflecting a rich and diverse chemical composition (Table 1). Among the identified metabolites, flavonoids constituted the most prominent class, followed by tannins, phenolic acids, triterpenoids, and coumarins. Subsequent UPLC-MS/MS analysis of SABE revealed the emergence of 14 modified metabolites (Table 1). These results highlight the enzymatic versatility of *A. niger* in catalysing substantial molecular modifications, thereby expanding the chemical landscape of the extract through targeted bioconversion.

### Comprehensive annotation of SAE and SABE metabolites via GNPS molecular networking

**Fig. 2:**
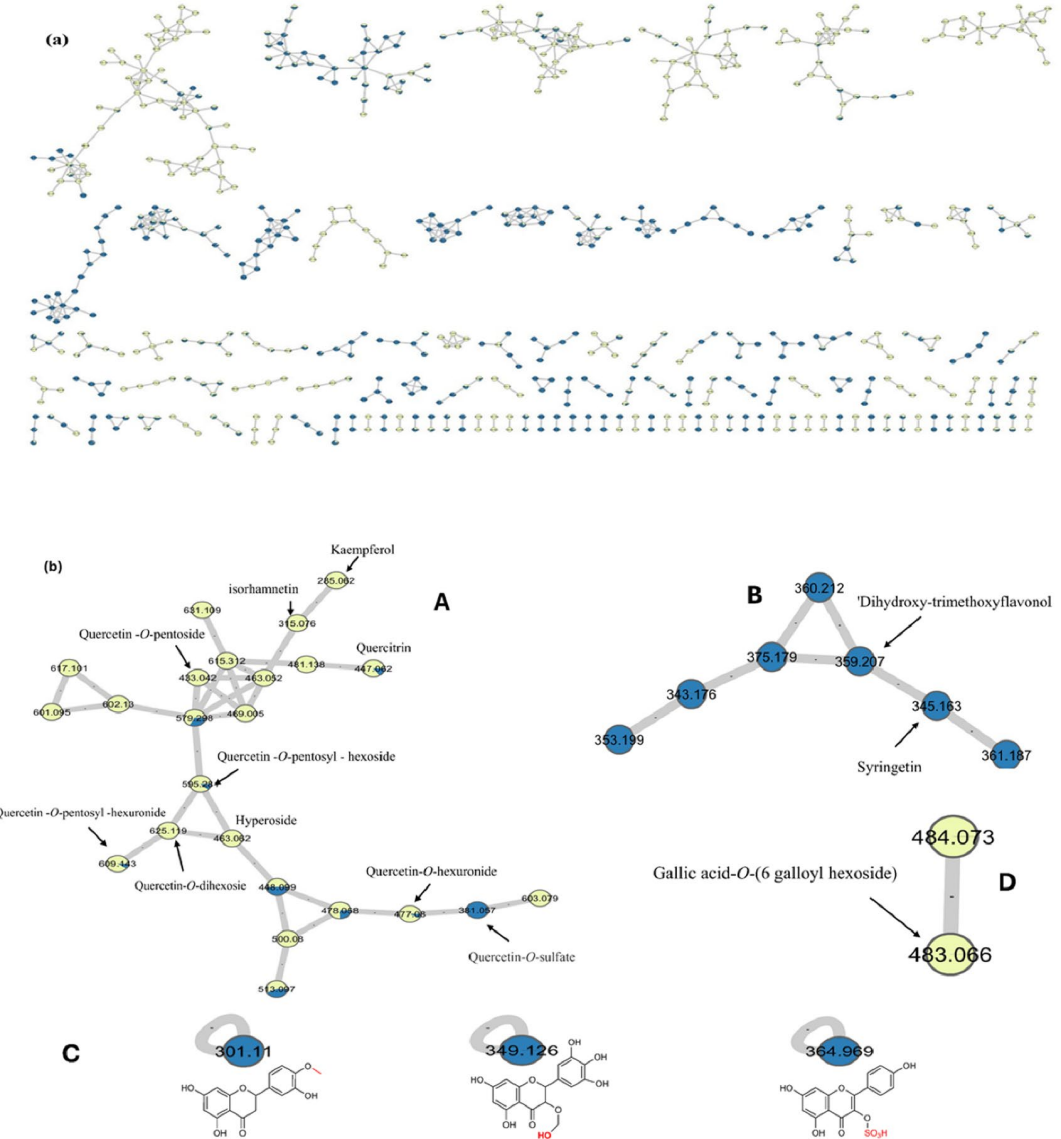
**(a)** Full molecular network created via UPLC-T-TOF-MS-MS data in negative ionization mode from SAE (yellow color) and SABE (blue color) and **(b)** expansion of major clusters showing microbial transformation effects on *S. australe* metabolites. Cluster A: quercetin derivatives; cluster B: syringetin and its microbial metabolite dihydroxy-trimethoxy flavonol; C: self-looped nodes of microbial metabolic products of *S. australe;* and cluster D: Gallic acid-*O*-(6 galloyl hexoside).

A major benefit of networking is its ability to identify known compounds along with their possible analogues^31^. This method was applied herein for the identification of SAE metabolites and their biotransformed metabolites via *A. niger* culture. The MN constructed from the MS/MS data of the analysed samples incorporated 369 interconnected nodes organized into 30 distinct clusters (Fig. 2a). Among these, two clusters were particularly noteworthy: cluster A, representing quercetin derivatives, while cluster B, corresponding to syringetin. Interestingly, the biotransformed metabolites on SABE appeared predominantly as self-looped nodes, suggesting that these unique metabolites presented limited structural similarity to other detected compounds (Fig. 2b).

**Table 1:** Tentatively identified metabolites via UPLC-T-TOF–MS/MS in the *S. australe* leaves extract (SAE) and *S. australe* biotransformed extract (SABE) in negative ionization mode.

M#	*R*_t_ (min)	Metabolites	Formula	[M-H]^−^	Error (PPM)	MS/MS	SAE	SABE	References
Flavonoids									
1.	4.16	Quercetin-*O*-di-hexoside	C_27_H_30_O_17_	625.1416	2.7	463, 301,179,151	√	-	^32^
2.	4.38	Myricetin-*O*-hexuronide	C_21_H_18_O_14_	493.0604	−1.8	317, 299,179, 151	√	√	^33^
3.	4.45	Quercetin-*O*-pentosyl-hexuronide	C_26_H_26_O_17_	609.1118	5.2	477, 301,179,151	√	√	^34^
4.	4.79	Pentahydroxy-3-hydroxymethoxy-flavanone	C_16_H_14_O_9_	349.0553	−0.3	331, 301, 299, 151	-	√	**-**
5.	4.80	Isorhamnetin-*O*-pentosyl-hexuronide	C_27_H_28_O_17_	623.0827	19.9	491, 315, 300,179	√	√	^34^
6.	4.81	Quercetin-*O-*pentosyl-hexoside	C_26_H_28_O_16_	595.1270	−2.7	463,301,179,151	√	-	^32^
7.	4.90	Delphinidin-*O*-deoxyhexosyl-hexoside	C_27_H_31_O_16_	609.1463	2.1	463, 301,287,179	√	-	^35^
8.	4.95	Quercetin-*O*-hexuronide	C_21_H_18_O_13_	477.0679	3.2	301, 273, 179, 151, 121	√	√	^32^
9.	5.13	Mearnsetin-*O*-hexuronide	C_22_H_20_O_14_	507.0760	−1.8	331, 316, 301, 179, 151	√	√	**-**
10.	5.41	Kaempferol-*O*-hexuronide	C_21_H_18_O_12_	461.0692	−4.9	285,257,189,151	√	-	^36^
11.	5.47	Quercetin-*O*-sulfate	C_10_H_10_O_10_S	380.9909	−0.5	301, 179, 151,121	-	√	-
12.	5.50	Isorhamnetin-*O*-hexuronide	C_16_H_12_O_7_	491.0820	0.0	315, 300, 179,163,151	√	-	^37^
13.	5.51	Taxifolin-*O*-hexoside	C_21_H_22_O_12_	465.0981	−10.1	303, 285, 179,151,107	√	-	^38^
14.	5.48	Eriodictyol-*O*-di-hexoside	C_27_H_32_O_16_	611.1480	−20.8	449, 287, 151,135	√	-	^34^
15.	5.80	Quercetin-*O*-hexosyl-hexuronide	C_27_H_28_O_18_	639.1200	1.3	477, 301, 179, 151	√	√	^34^
16.	6.06	Myricetin-*O*-pentoside	C_20_H_18_O_12_	449.0796	18.1	317, 299,179, 151	√	-	^39^
17.	6.07	Myricetin-*O*-deoxyhexoside	C_21_H_20_O_12_	463.0860	−2.4	317, 299,179, 151	√	-	^39^
18.	6.08	Mearnsetin-*O*-hexosyl-hexuronide	C_28_H_30_O_19_	669.1328	4.6	507, 331, 301,179,151	√	-	^34^
19.	6.09	Syringetin	C_17_H_14_O_8_	345.0637	9.4	330, 314, 299, 285, 151	-	√	^40^
20.	6.15	Gossypin	C_21_H_20_O_13_	479.0807	−2.8	317, 299, 271, 195, 193, 123	√	-	^41^
21.	6.17	Kaempferol-*O*-sulfate	C_15_H_10_O_9_S	364.9993	0.3	285, 151,133,124.108	-	√	**-**
22.	6.40	Mearnsetin-*O*-hexoside	C_22_H_22_O_13_	493.0972	−0.1	331, 316, 301, 179, 151	√	-	^42^
23.	6.44	Isorhamnetin-*O*-sulfate	C_16_H_12_O_10_S	395.0044	−5.9	315, 300,179,163,151	-	√	**-**
24.	6.50	Isorhamnetin-*O*-hexosyl-hexuronide	C_28_H_30_O_18_	653.1265	−12.8	491, 315, 301,179,151	√	√	^34^
25.	6.69	Okanin-*O*-hexoside	C_21_H_22_O_11_	449.1105	5.9	287, 269, 151, 135	√	-	^43^
26.	6.73	Kaempferol-*O*-deoxyhexosyl-hexoside	C_27_H_30_O_15_	593.1028	−4.9	447, 285,151,133,108	√	-	^44^
27.	6.74	Quercetin-*O*-pentoside	C_20_H_18_O_11_	433.0763	−0.4	301, 179, 151,121,107	√	-	^32^
28.	6.75	Quercetin -*O*-pentosyl -deoxyhexoside	C_26_H_28_O_15_	579.1345	0.1	447,301,179,151	√	√	^34^
29.	6.76	Eriodictyol-*O*-hexoside	C_21_H_22_O_11_	449.1063	−3.4	287, 269, 151,135	√	-	^32^
30.	7.03	Quercitrin	C_21_H_20_O_11_	447.0949	6.1	301, 255, 179, 151, 121	√	√	^32^
31.	7.17	Isorhamnetin-*O*-hexoside	C_22_H_22_O_12_	477.1020	0.3	315, 300, 163, 151	√	-	^45^
32.	7.19	Taxifolin-*O*- deoxyhexoside	C_21_H_22_O_11_	449.1003	−16.7	303, 179, 151, 107	√	√	-
33.	7.39	Hyperoside	C_21_H_20_O_12_	463.0895	5.2	301, 271,179, 151	√	-	^46^
34.	7.54	Isorhamnetin-*O*-deoxyhexosyl-hexoside	C_28_H_32_O_16_	623.1508	−15.8	477, 315, 285,179,151	√	√	^45^
35.	7.62	Quercetin-*O*-hexoside	C_21_H_20_O_12_	463.0841	−6.5	301, 179, 151,107	√	-	^39^
36.	7.63	Hesperidin	C_28_H_34_O_15_	609.1794	−3.3	301, 286, 242,163	√	-	^47^
37.	7.64	Naringenin-*O*-hexoside	C_21_H_22_O_10_	433.1093	−8.4	271, 177, 151, 119	√	-	^32^
38.	7.76	Mearnsetin-*O*-sulfate	C_16_H_12_O_11_S	411.0044	6.6	331, 316, 206.9, 151	-	√	**-**
39.	7.77	Kaempferol-*O*-deoxyhexoside	C_21_H_20_O_10_	431.0950	−5.0	285, 257,124.108	√	√	^48^
40.	7.80	Myricetin	C_15_H_10_O_8_	317.0290	−0.6	271, 179, 165, 151, 125, 107	√	√	^49^
41.	7.87	Isorhamnetin-*O*-deoxyhexoside	C_22_H_22_O_11_	461.1001	0.6	315, 300, 179,151	√	-	^50^
42.	8.57	Taxifolin	C_15_H_12_O_7_	303.0510	3.5	179,151, 123,107	√	√	^41^
43.	8.62	Tetrahydroxyflavanone	C_15_H_12_O_6_	287.0569	6.6	181,151, 135, 107	√	√	-
44.	8.72	Methyl myricetin	C_16_H_12_O_8_	331.0489	12.4	316, 224, 207, 179, 124	√	-	^51^
45.	8.97	Mearnsitrin	C_22_H_22_O_12_	477.1433	−4.3	331, 301, 179, 151	√	√	^32^
46.	9.02	Trihydroxy-dimethoxy flavonol	C_17_H_14_O_7_	329.0610	9.9	314,299, 298, 283, 177,149	-	√	-
47.	9.14	Quercetin	C_15_H_10_O_7_	301.0330	−1.9	273, 193, 179, 151, 121, 107	√	√	^52^
48.	9.15	Mearnsetin	C_16_H_12_O_8_	331.0448	−0.1	316, 300, 179, 152, 151, 107	√	√	^51^
49.	9.75	Naringenin	C_15_H_12_O_5_	271.0623	8.1	177, 151, 119, 107	√	√	^44^
50.	9.86	Hesperetin	C_16_H_14_O_6_	301.1193	0.6	286, 193, 179, 151, 149, 108	-	√	-
51.	10.32	Apigenin	C_15_H_10_O_5_	269.0422	−8.3	229,207,176, 117	√	√	^53^
52.	10.38	Luteolin	C_15_H_10_O_6_	285.0377	−5.8	267, 175, 151, 133, 121	√	√	^52^
53.	10.50	Kaempferol	C_15_H_10_O_6_	285.0300	1.5	257, 151, 133, 124, 108, 93	√	-	^52^
54.	10.78	Trihydroxy-methoxy flavonol	C_16_H_12_O_7_	315.0506	2.1	300, 179,164, 151	√	-	^45^
55.	10.92	Butein	C_15_H_12_O_5_	271.0600	9	253, 165,227, 135	-	√	-
56.	11.98	Dihydroxy-trimethoxy flavonol	C_18_H_16_O_8_	359.0992	5.4	344, 329, 313, 251, 180.9	-	√	-
57.	15.83	Tetrahydroxy-methoxy flavone	C_16_H_12_O_7_	315.0536	11.7	300, 285,165, 121	√	√	^32^
58.	17.92	Trihydroxy-methoxy flavone	C_16_H_12_O_6_	299.0530	−6.7	284, 253,151,107	√	√	^48^
**Coumarins**									
59.	3.54	Bergenin	C_14_H_16_O_9_	327.0672	−11.8	312, 299, 281, 259, 192	√	-	^54^
60.	4.59	Dihydroxycoumarin	C_9_H_6_O_4_	177.0199	9.4	149, 133, 107,93,89	√	-	^35^
**Gallotannins**									
61.	1.19	Gallic acid	C_7_H_6_O_5_	169.0131	−0.3	125, 124,97,79	√	√	^32^
62.	1.38	Glucogallic acid	C_13_H_16_O_10_	331.0672	3.7	169, 151, 125,97,79	√	-	^55^
63.	3.42	Gallic acid-*O*-(6 galloyl hexoside)	C_20_H_20_O_14_	483.0777	1.7	331, 313, 271, 169, 151	√	-	^55^
64.	4.41	Methyl gallate	C_8_H_8_O_5_	183.0300	10.4	168, 156, 139, 124	√	√	^32^
65.	5.25	Tetra-*O* -galloyl -hexose	C_34_H_28_O_22_	787.0918	9.0	635, 483, 169,125	√	-	**-**
**Ellagitannins**									
66.	1.29	Hexahydroxydiphenoyl-hexoside	C_20_H_18_O_14_	481.0601	−2.6	301, 275,275,169	√	-	^55^
67.	1.39	Dihexahydroxydiphenoyl-hexoside	C_34_H_24_O_22_	783.0674	0.1	481, 301, 275, 169	√	-	^55^
68.	3.57	Casuarictin	C_41_H_28_O_26_	935.0728	−6.1	783,633, 301, 169	√	-	^55^
69.	5.20	Digalloyl-hexahydroxydiphenoyl-hexoside	C_34_H_28_O_26_	785.1982	2.0	633, 615,301, 275, 169	√	-	^55^
70.	5.69	Hexahydroxydiphenoyl-galloyl-hexose	C_27_H_22_O_18_	633.0733	1.7	481,301, 275,169	√	-	^55^
71.	8.46	Ellagic acid	C_14_H_6_O_8_	300.9995	5.3	273, 257, 229,201,188	√	√	^55^
**Phenolic and organic acids**									
72.	0.86	Citric acid	C_6_H_8_O_7_	191.0188	0.8	173, 147, 129, 111, 103	-	√	-
73.	0.95	Isopropylmalic acid	C_7_H_12_O_5_	175.0579	−10.9	157, 131,113,87	-	√	-
74.	1.01	Malic acid	C_4_H_6_O_5_	133.0139	5.6	115, 87,89,73, 71	√	-	^56^
75.	1.03	Tartaric acid	C_4_H_6_O_6_	149.0102	14.3	130.9, 105, 103, 87	√	√	^57^
76.	1.09	Maleic acid	C_4_H_4_O_4_	115.0025	−0.7	87, 83,71, 69	√	-	^56^
77.	1.11	Glyceric acid	C_3_H_6_O_4_	105.0171	−10.8	87, 75,72.9,60,59	√	-	-
78.	1.15	Methylglutaric acid	C_6_H_10_O_4_	145.0602	8.7	127, 101,99,85,83	-	√	-
79.	1.20	Benzoic acid	C_7_H_6_O_2_	121.0276	−6.7	106,93, 80,77	√	√	^58^
80.	1.29	Dihydroxybenzoic acid -*O*-hexoside	C_13_H_16_O_9_	315.0712	0.4	153, 135, 109,108,83,71	√	-	^55^
81.	1.31	Citraconic acid	C_5_H_6_O_4_	129.0177	−4.1	111, 101, 85, 67	√	√	-
82.	1.34	Sinapic acid	C_11_H_13_O_5_	223.0600	−0.4	208, 193, 179, 177, 164, 149	√	-	^59^
83.	1.41	Dihydroxybenzoic acid	C_7_H_6_O_4_	153.0179	−2.2	135, 109,85,79,71	√	√	^57^
84.	3.74	Hydroxybenzoic acid	C_7_H_6_O_3_	137.0248	10.8	108, 93,73,66	√	-	^57^
85.	4.94	Catechol	C_6_H_6_O_2_	109.0289	4.8	91, 77, 67, 65	-	√	-
86.	6.60	Vanillic acid	C_8_H_8_O_4_	167.0356	11.9	152, 123, 108,83,61,60	√	√	^60^
87.	6.66	Homogenentisic acid	C_8_H_8_O_4_	167.0356	11.5	122.9, 108,83,61,60	√	-	^57^
88.	7.98	Phlorizin	C_21_H_24_O_10_	435.1200	−9.4	273, 205, 189, 151	√	-	^32^
**Triterpenoids**									
89.	10.63	Cleistocalyxic acid D	C_30_H_46_O_5_	485.3274	2.6	441, 409,397,278	√	-	-
90.	11.80	Cleistocalyxic acid I	C_30_H_46_O_6_	501.3210	0.2	483, 471,457,278	√	√	-
91.	12.55	Cleistocalyxic acid E	C_30_H_48_O_6_	503.3370	0.6	485,467, 455,441,409	√	-	-
92.	18.91	Arjunolic acid	C_30_H_48_O_5_	487.3447	5.9	469, 441, 425,203	√	-	^61^
93.	19.83	Oleanolic acid	C_30_H_48_O_3_	455.3540	5.9	437, 411, 409,249	√	√	^61^
94.	24.29	Maslinic acid	C_30_H_48_O_4_	471.3481	2.6	453, 427,409,249	√	√	^61^

#### Flavonoids

The MS/MS fragmentation patterns of the flavonoid derivatives revealed the characteristic loss of hexosyl, deoxyhexosyl, and hexuronyl residues, corresponding to neutral losses of 162, 146, and 176 Da, respectively^34^. These fragmentation patterns were observed for metabolites such as quercetin-*O*-hexoside (**M35**) (Fig. S2), kaempferol-*O*-deoxyhexoside (**M39**) and myricetin-*O*-hexuronide (**M2**) (Fig. S3). The fragmentation processes were followed by Retro-Diels-Alder (RDA) fragmentation, a well-established pathway that further aids in the structural elucidation of flavonoid aglycones^62^.

#### Gallotannins

Gallotannins are characterized by their distinctive fragmentation behavior, enabling precise structural characterization. Their spectra are marked by the predominant formation of fragment ions [M-H-170]^-^ and [M-H-152]^-^, which correspond to the neutral loss of gallic acid and galloyl residues, respectively. For example, gallic acid-*O*-(6-galloyl hexoside) (**M63)** (Fig. S4) was identified on the basis of its deprotonated molecular ion at *m/z* 483.0777, and its MS2 spectrum was attributed to the loss of a galloyl moiety, resulting in a key fragment ion at *m/z* 331.0669 and a fragment ion at *m/z* 313.0584, corresponding to the neutral loss of gallic acid. Similarly, tetra-*O*-galloyl-hexose (**M65)** (Fig. S5) exhibited a deprotonated ion at *m/z* 787.0918 and subsequent loss of 2 galloyl moieties, as confirmed by the 635.0852 and 483.0757 fragments.

#### Ellagitannins

Ellagitannins are characterized mainly by galloyl and HHDP moieties, which results in the detection of characteristic neutral losses, including galloyl, gallic acid and HHDP. Additionally, a prominent fragment ion at *m/z* 301 was observed, originating from the lactonization of the HHDP ester group into the more stable ellagic acid structure, and was the most detected among these metabolites. Casuarictin (**M68**) (Fig. S6) was detected with a deprotonated molecular ion at *m/z* 935.0728. Fragmentation of **M68** produced an ion at *m/z* 783.0683, indicative of the neutral loss of a galloyl moiety, whereas the subsequent formation of a key ion at *m/z* 301.0073 reflected the conversion of the HHDP group into a stable ellagic acid structure. Similarly, di-galloyl-HHDP-hexoside (**M69**, Fig. S7) was identified with a deprotonated molecular ion at *m/z* 785.1982. Its MS2 fragmentation profile revealed an ion at *m/z* 633.0651, indicating the loss of a galloyl group, followed by the generation of a fragment ion at *m/z* 301.0071 due to HHDP lactonization.

#### Phenolic acids

The MS2 fragmentation of phenolic acids revealed typical losses of 18 Da (H₂O), 44 Da (CO₂), and 62 Da (H₂O and CO₂). For instance. sinapic acid (**M82)** (Fig. S8) was identified by its deprotonated ion at *m/z* 223.0600, with fragment ions at *m/z* 208.0378 [M-H–CH_3_]^-^ and *m/z* 179.0547 [M-H-CO_2_]^-^. Vanillic acid (**M86)** (Fig. S9) was identified by its deprotonated ion at *m/z* 167.0359, and further deprotonation in MS2 resulted in a fragment ion at *m/z* 122.9670, indicating the loss of CO_2_.

#### Triterpenoids

In pentacyclic triterpenoid acids, the predominant fragmentation pathways typically begin with the loss of neutral molecules such as H₂O or CO₂, followed by characteristic cleavages within the ring system. Notably, RDA cleavage of ring C serves as a key diagnostic feature in the mass spectra of pentacyclic triterpenoid acid derivatives, particularly those bearing carboxyl functional groups in rings D or E^63^. For example, oleanolic acid (**M93**) (Fig. S10) exhibited a deprotonated molecular ion at *m/z* 455.3546. The [M − H]^-^ ion underwent subsequent fragmentation in MS2, yielding fragment ions at *m/z* 437.1867 and *m/z* 411.0034, corresponding to the successive losses of H₂O and CO₂, respectively. The RDA cleavage of ring C generates a fragment ion at *m/z* 248.9605, representing a moiety comprising rings D and E along with a portion of ring C. Similarly, maslinic acid (**M94**) (Fig. S11) showed a deprotonated molecular ion at *m/z* 471.3481, with a fragment ion at *m/z* 453.1831 arising from the loss of H₂O. Further fragmentation results in the formation of an ion at *m/z* 409.3057 due to an additional loss of CO_2_. The characteristic RDA cleavage of ring C was observed at *m/z* 248.9613, confirming the structural integrity of the D and E rings.

### GNPS-assisted prediction of the metabolites of *S. australe* biotransformed by *A. niger*

Clear distinctions between the SAE and SABE samples were apparent upon visual observation of the MNs. Initial analysis of the network revealed both qualitative and quantitative differences in the metabolite profiles of SAE and SABE.

#### Metabolite biotransformation

Flavonols, a prominent class of metabolites in *S. australe*, appeared to undergo significant sulfonation when exposed to *A. niger* culture. Among these, quercetin stood out in SABE, alongside its sulfonated metabolite, quercetin-*O*-sulfate. As revealed through the MN (Fig. 2b). Quercetin-*O*-sulfate (**M11)** (Fig. S12) exhibited a molecular ion at *m/z* 380.9909, and fragmentation led to a major ion at *m/z* 301.0329, indicating the neutral loss of the sulfate group (80 Da)^64^. Furthermore, two distinct aglycone fragments characteristic of the RDA fragmentation pattern were detected. The suggested mechanism for this sulfonation process is likely catalyzed by sulfotransferase enzymes produced by *A. niger*^65^.

Moreover, isorhamnetin undergoes selective methylation at the 4’ position on the ring B when incubated with *A. niger* culture. This transformation results in the formation of trihydroxy-dimethoxy flavonol (**M46)** detected at *m/z* 329.0688 in SABE. The fragmentation pattern revealed key ions at *m/z* 298 and 283. The suggested mechanism behind these methylation reactions likely involves methyltransferase enzymes produced by *Aspergillus* species^10^.

Metabolite **4** (Pentahydroxy-3-hydroxymethoxy-flavanone) was identified by its deprotonated ion at *m/z* 349.0553 (Fig. S13). Fragmentation revealed a key ion at *m/z* 331 due to the loss of an H_2_O molecule and a fragment at *m/z* 301 from subsequent methoxy cleavage. The biosynthesis of this compound begins with the enzymatic hydroxylation of the methoxy group at position 3 on ring C of 3-*O*-methylmyrecitin, which is mediated by the hydroxylase enzyme. This is followed by the reduction of ring C via reductase enzymes of *Aspergillus*, resulting in hydrogenation and stabilization of the chroman structure^66^.

Following incubation with *A. niger* culture, most glycosides in the SAE were no longer detectable, suggesting that extensive hydrolysis of their aglycon was mediated by microbial glycosidase enzymes^67^. This hydrolysing activity was confirmed through MN analysis, where glycosides such as quercetin-*O*-dihexoside (**M1**) and hyperoside (**M33**) were identified in SAE but were absent in SABE.

*A. niger* is an effective source for producing tannase enzymes^68^. Tannin acyl hydrolase (Tannase) is a vital enzyme that can hydrolyse ester bonds (galloyl esters of alcohols) and depside bonds (galloyl esters of gallic acid). It typically acts on the C–O and ester linkages found in hydrolysable tannins, resulting in biotransformed products such as gallic acid and glucose. For ellagitannins, tannase selectively targets galloyl groups, resulting in the degalloylation of ellagitannins and the formation of biotransformed products such as ellagic acid^69^. In the present study, the biotransformation of gallotannins was monitored through the detection of cluster D (gallic acid-*O*-galloyl hexoside), a precursor compound present before biotransformation. This compound was converted into gallic acid following enzymatic hydrolysis, which aligns with previous research on tannase-catalyzed reactions. Furthermore, hydrolysis of ellagitannins was observed, leading to the production of ellagic acid, which further corroborates the specificity of the enzyme for galloyl units within ellagitannin structures.

The production of citric acid by *A. niger* is attributed to its highly efficient central carbon metabolism, which primarily involves glycolysis and the tricarboxylic acid (TCA) cycle. During the biotransformation process, sugars such as glucose are metabolized through glycolysis, yielding pyruvate as a key intermediate. Pyruvate is subsequently converted into acetyl-CoA and enters the TCA cycle. This metabolic bottleneck resulted in the intracellular accumulation of citric acid. Citric acid is subsequently transported out of the mitochondria and secreted into the extracellular environment through specialized transport mechanisms^70^.

### Multivariate analysis

**Fig. 3:**
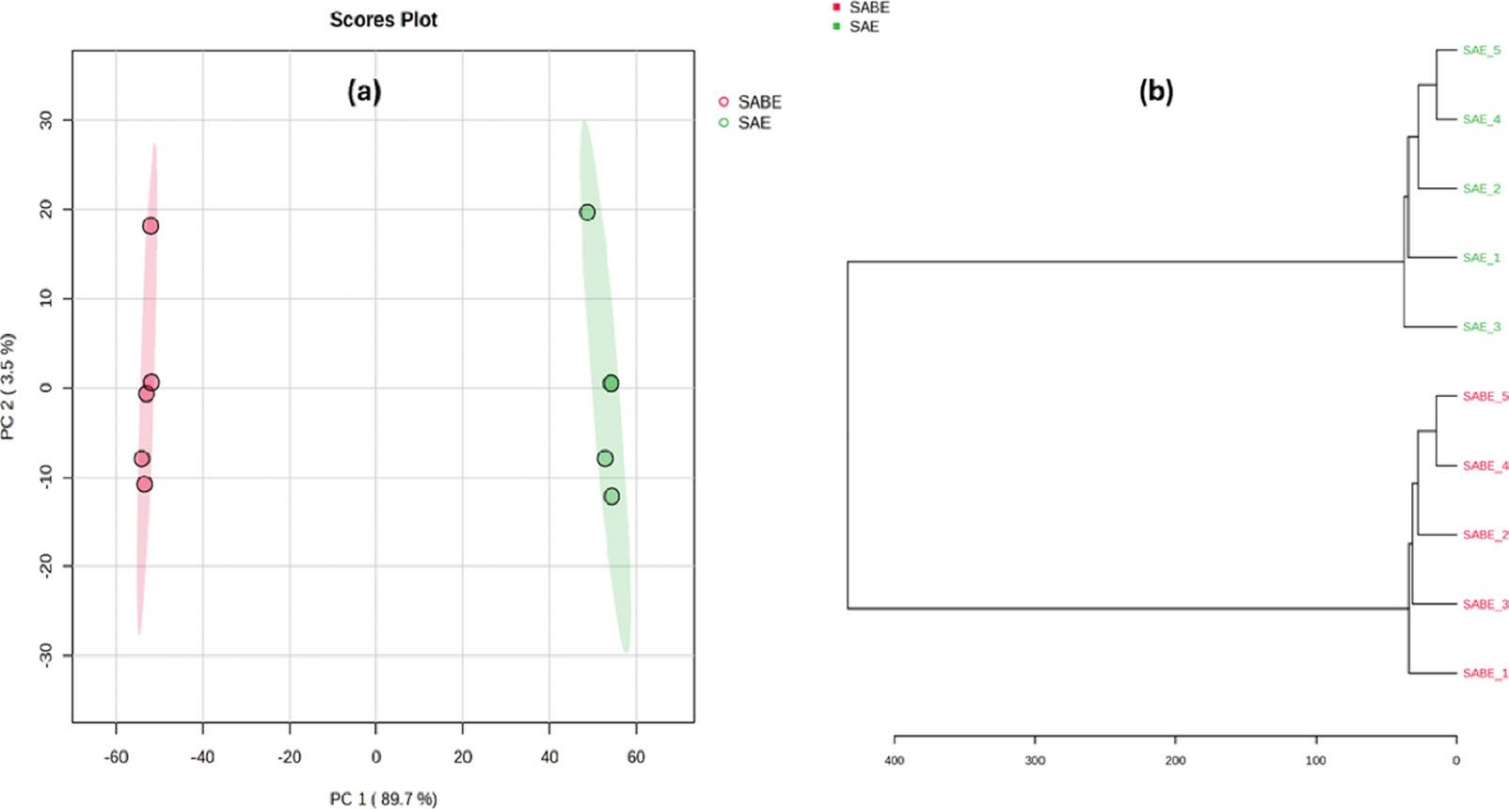
Multivariate analysis of metabolomic profiles illustrating sample clustering and variation: (**a**) unsupervised PCA score plot demonstrating the distribution and discrimination of samples based on their metabolic profiles.; (**b**) HCA dendrogram showing grouping patterns among samples.

Unsupervised PCA was initially employed to explore the intrinsic variation within the LC–MS dataset and to assess the impact of biotransformation on the metabolic profile of *S. australe* extract. As illustrated in Fig. 3a, a clear and well-defined separation between the SAE and SABE samples was achieved via the first principal component (PC1), which accounted for 89.7% of the total variance, whereas PC2 explained an additional minor proportion (~ 3.5%). The high cumulative explained variance (R²X = 0.932) reflects the robustness of the model. Notably, the tight clustering of replicates within each group indicates excellent analytical reproducibility and minimal intragroup variability, confirming the reliability of the dataset. The observed separation highlights the substantial metabolic reprogramming induced by *A. niger* biotransformation. This clustering pattern was further supported by HCA (Fig. 3b), which distinctly segregated the samples into two major clusters corresponding to the SAE and SABE groups. The absence of overlap between clusters, combined with strong intragroup similarity, reinforces the presence of significant biochemical divergence between the two conditions.

**Fig. 4:**
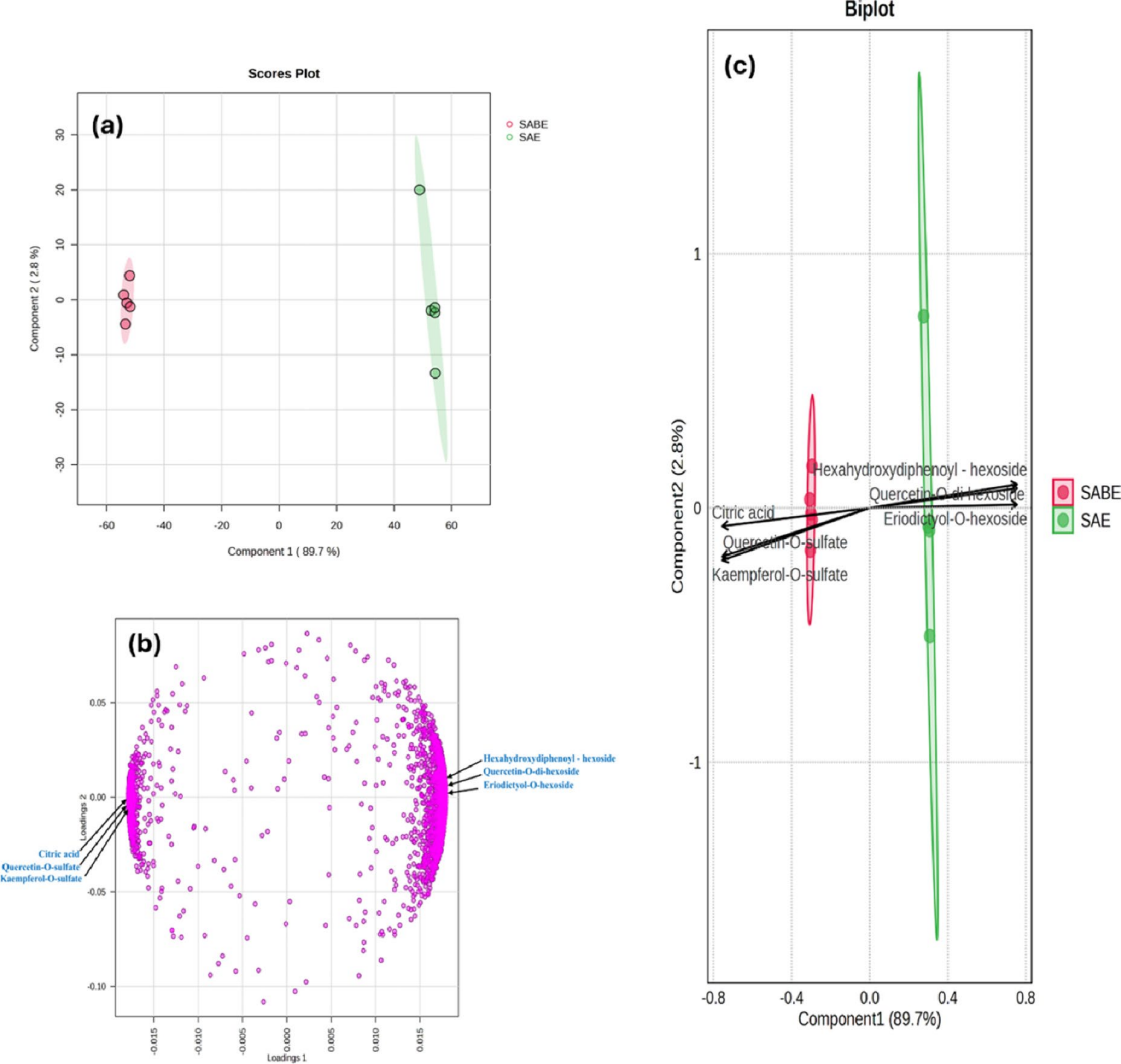
Supervised PLS-DA of metabolomic profiles: (**a**) PLS-DA score plot illustrating the discrimination between SAE and SABE samples; (**b**) loading plot showing metabolite contributions to model components; variables farther from the origin have the strongest influence on class discrimination; (**c**) PLS-DA biplot representing the relationship between samples and discriminant metabolites.

To further enhance class discrimination and identify metabolites responsible for group separation, supervised PLS-DA was conducted. The PLS-DA score plot (Fig. 4a) demonstrated complete separation with no overlap between the SAE and SABE samples, with Component 1 and Component 2 explaining 89.6% and 2.8% of the variance, respectively. The model exhibited strong predictive ability, as indicated by a positive Q² value, confirming its robustness and reliability. Interpretation of the PLS-DA loading plot and biplot (Fig. 4b, c) respectively revealed key metabolites driving this separation. Specifically, the SABE samples were predominantly associated with citric acid, quercetin-*O*-sulfate, and kaempferol-*O*-sulfate, whereas the SAE samples were characterized by relatively high levels of hexahydroxydiphenoyl-hexoside, quercetin-*O*-dihexoside, and eriodictyol-*O*-hexoside. These metabolites represent major contributors to the biochemical distinction between SAE and SABE.

The supervised OPLS-DA model further refined class discrimination by removing orthogonal variation unrelated to class separation. The model demonstrated excellent statistical performance (R²X = 0.896, R²Y = 0.999, and Q² = 0.998), alongside a significant CV-ANOVA (*p* = 0.008), confirming model validity. Importantly, the minimal difference between R²Y and Q² indicates no evidence of model overfitting, which is further supported by permutation testing (Fig. S14).

**Fig. 5:**
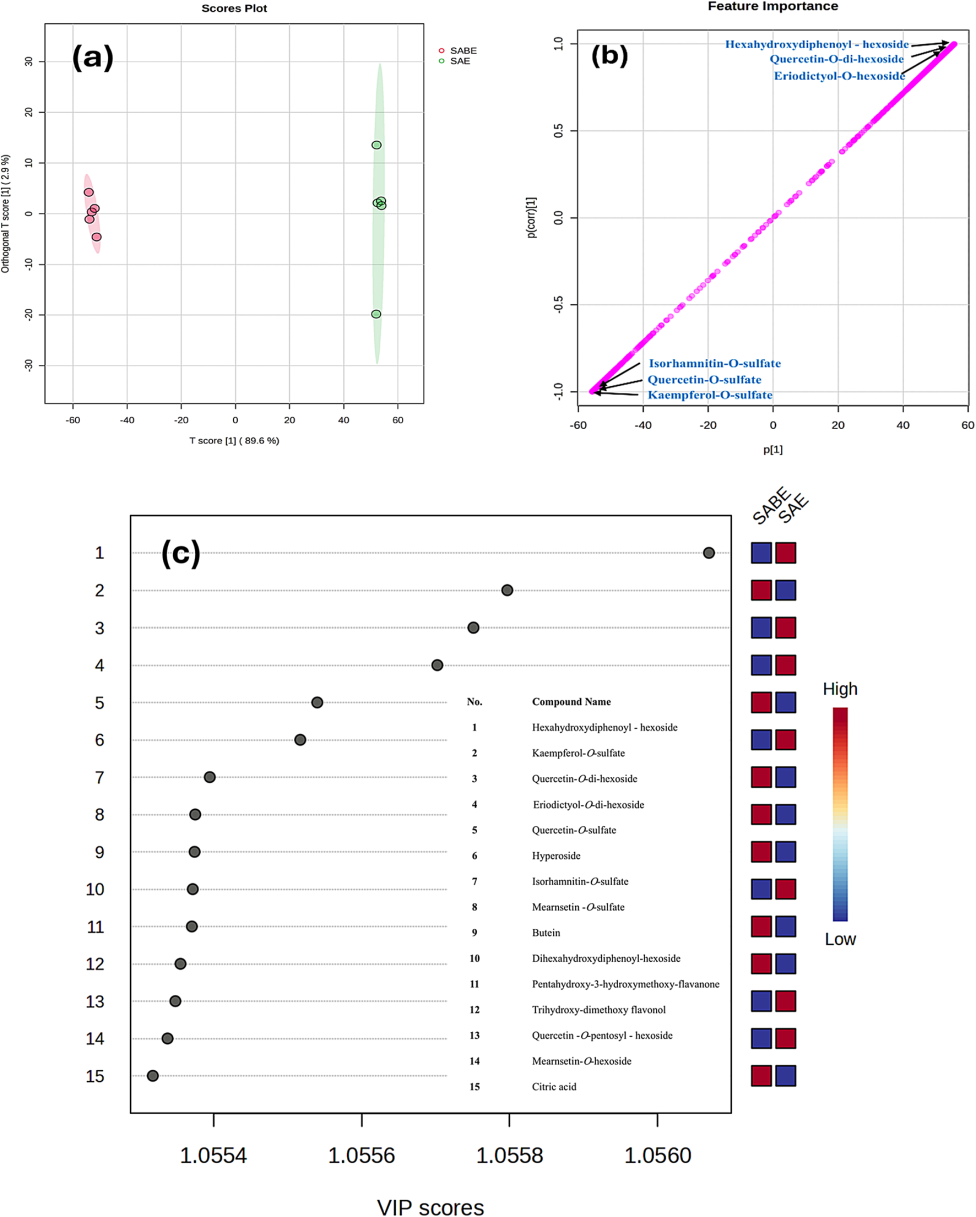
Ortho PLS-DA-based metabolomic differentiation of SAE and SABE: (**a**) Score plot showing robust separation between SAE and SABE samples; (**b**) S-plot highlighting metabolites with the strongest contributions to class discrimination; (**c**) VIP analysis identifying 15 key metabolites as putative biomarkers in SAE versus SABE, with red denoting high dominance and blue indicating low dominance.

The corresponding S-plot enabled visualization of variables with both high covariance and correlation. Metabolites such as hexahydroxydiphenoyl-hexoside, quercetin-*O*-dihexoside, and eriodictyol-*O*-hexoside were located in the upper-right quadrant, indicating a strong positive correlation with SAE samples. In contrast, citric acid, quercetin-*O*-sulfate, and kaempferol-*O*-sulfate were positioned in the lower-left quadrant, reflecting their association with SABE samples. These compounds can be considered robust discriminant biomarkers reflecting the biochemical consequences of fungal biotransformation. Further confirmation was obtained through VIP analysis (Fig. 5c), where all selected metabolites presented VIP scores > 1.0, indicating their significant contribution to class separation and model construction.

**Fig. 6:**
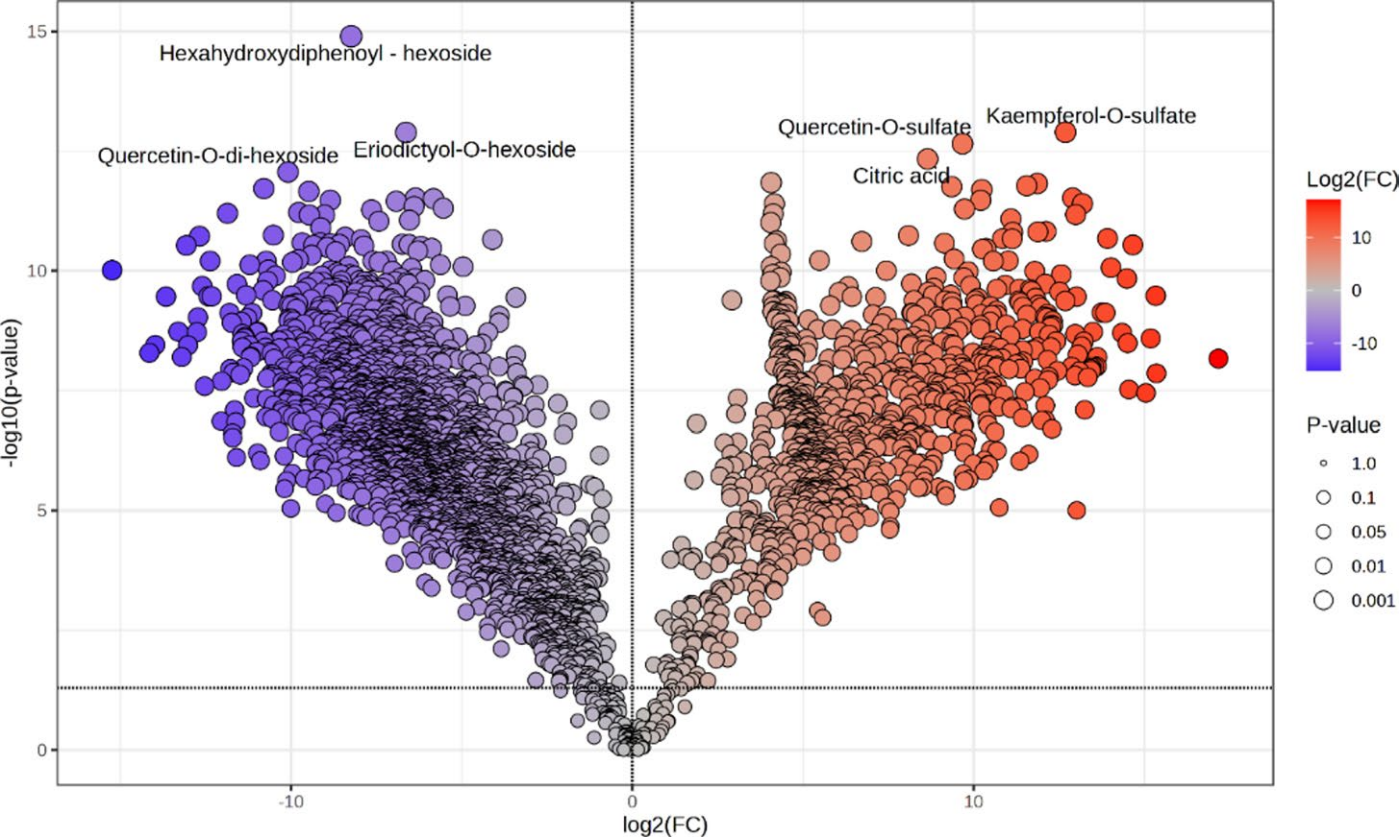
The volcano plot shows the differentially abundant metabolite expression levels in SAE and SABE samples. Red, blue, and gray dots indicate upregulated, downregulated, and nonsignificantly differentially expressed metabolites, respectively.

Finally, a volcano plot (Fig. 6) was generated to assess univariate statistical significance and fold-change magnitude. The analysis revealed a large-scale metabolic shift, with 977 features significantly upregulated and 2,358 features significantly downregulated in SAE relative to SABE (based on defined thresholds of log₂FC and *p*-value). Notably, metabolites such as hexahydroxydiphenoyl-hexoside and quercetin-*O*-dihexoside were markedly upregulated in SAE, whereas citric acid and flavonoid sulfates were enriched in SABE. Overall, the integration of unsupervised, supervised, and univariate analyses provides compelling evidence of profound metabolomic reprogramming induced by *A. niger* biotransformation, highlighting specific metabolite classes (phenolic glycosides vs. sulfated flavonoids) as key biochemical signatures.

### ABTS and DPPH-based in vitro antioxidant screening

**Fig. 7:**
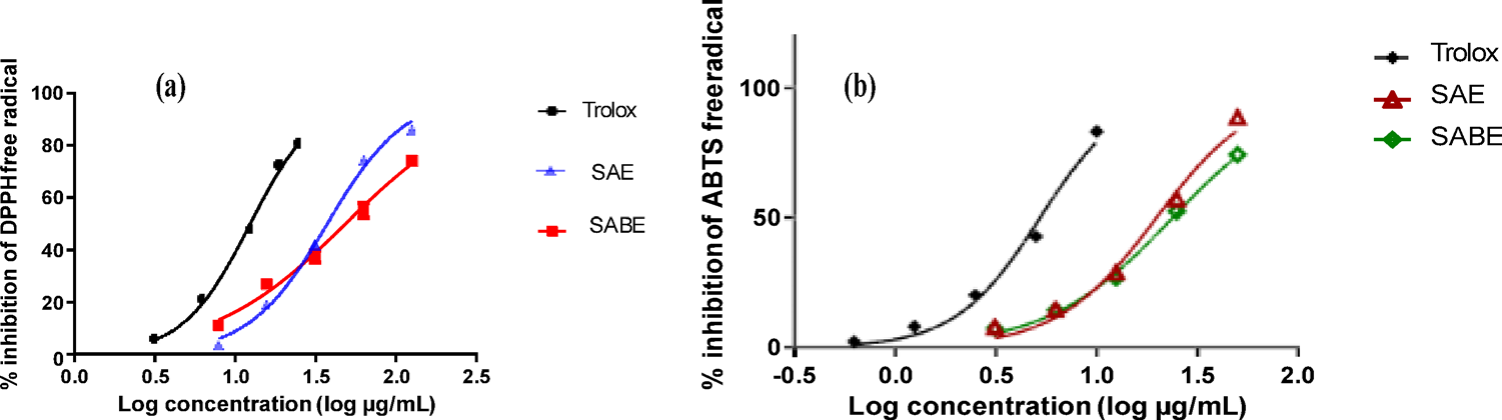
Percent inhibition of (**a**) DPPH and (**b**) ABTS free radicals by SAE, SABE, and Trolox standard.

The antioxidant activities of SAE and SABE were comprehensively evaluated via two complementary assays; DPPH and ABTS. The dose-dependent scavenging activities of SAE and SABE are illustrated in **(**Fig. 7**).** SAE and SABE exhibited strong free radical neutralization, as indicated by a progressive increase in percentage inhibition with increasing concentration. This trend reflects a marked reduction in absorbance in the presence of the extracts, underscoring their significant antioxidant capacity mediated by electron or hydrogen atom donation.

In the DPPH assay, SAE exhibited IC₅₀ value of 36.96 ± 1.20 *µ*g/mL (*n* = 3), highlighting its potent radical-scavenging ability. In comparison, SABE demonstrated a higher IC₅₀ value of 48.50 ± 2.10 *µ*g/mL, indicating slight reduction of antioxidant activity after biotransformation. The standard antioxidant Trolox showed a markedly lower IC₅₀ value (12.32 ± 1.01 *µ*g/mL), confirming its strong antioxidant potential. Similarly, in the ABTS assay, SAE exhibited a lower IC₅₀ value (19.80 ± 0.85 *µ*g/mL) than SABE (23.79 ± 1.10 *µ*g/mL), indicating stronger antioxidant activity before biotransformation. Trolox showed the highest activity, with an IC₅₀ value of 5.23 ± 1.03 *µ*g/mL, confirming its effectiveness as a reference antioxidant. The SAE contained a high level of tannins; however, during microbial biotransformation, these compounds underwent hydrolysis. This degradation likely explains the observed reduction in the antioxidant activity of the SABE.

### In vitro evaluation of the inhibition of α-glucosidase, α-amylase, and pancreatic lipase enzymes

Table 2 reveals that SAE has a significant α-glucosidase inhibitory activity, surpassing the reference standard drug, along with moderate inhibitory effects on α-amylase and lipase. Upon microbial biotransformation with *A. niger*, the lipase inhibitory activity of SAE improved substantially, where the percent inhibition increased from 47.98 ± 1.73% to 74.49 ± 4.80% at 500 *µ*g/mL. For α-amylase at 500 *µ*g/mL, the percentages of inhibition showed minimal variation after biotransformation, ranging from 81.95 ± 0.59% to 79.93 ± 2.69%. However, a marked reduction in α-glucosidase inhibitory activity was observed, where the inhibition decreased from 58.84 ± 1.28% to 54.41 ± 1.64% at 1000 *µ*g/mL following microbial transformation.

**Table 2:** Effects of *S. australe* extract (SAE) and its biotransformed extract (SABE) on lipase, α-amylase, and α-glucosidase enzymes.

Samples	Inhibition %					
	Lipase		α-amylase		α-glucosidase	
	50* µ*g/mL	500* µ*g/mL	50 *µ*g/mL	500 *µ*g/mL	100 *µ*g/mL	1000 *µ*g/mL
SAE	38.66 ±3.66	47.98 ±1.73	19.29 ±0.41	81.95±0.59	58.47 ±0.38	58.84±1.28
SABE	39.77 ±3.13	74.49±4.80	19.69±0.77	79.93±2.69	48.21 ±2.70	54.41±1.64
Standard drugs	Orlistat		Acarbose		Acarbose	
	0.0001 *µ*g/mL	0.01 *µ*g/mL	7.8* µ*g/mL	125* µ*g/mL	62.5* µ*g/mL	250 *µ*g/mL
	29.76 ±2.47	59.48 ±5.41	37.80 ±2.48	92.29 ±0.32	34.67 ±2.30	56.13±3.94

The notable inhibitory activities of SAE and SABE against α-glucosidase and α-amylase are likely attributable to their high content of polyphenolic compounds, which are known for their enzyme-modulating properties. Previous studies have demonstrated that polyphenols can inhibit carbohydrate-digesting enzymes through specific interactions with catalytic or allosteric residues within their active sites, thereby interfering with substrate binding or catalysis^71^. Additionally, glycosylated phenolics and tannins may act as competitive inhibitors, mimic natural substrates of α-glucosidase, and thus reduce enzymatic activity^72^. Interestingly, a slight reduction in α-glucosidase inhibition following fungal biotransformation may be explained by the enzymatic hydrolysis of glycosides and tannins. The partial hydrolysis or degradation of tannins during biotransformation could therefore contribute to the observed decline in the inhibitory potency of the SABE.

### Statistical analysis of in-vitro bioassays

**Fig. 8:**
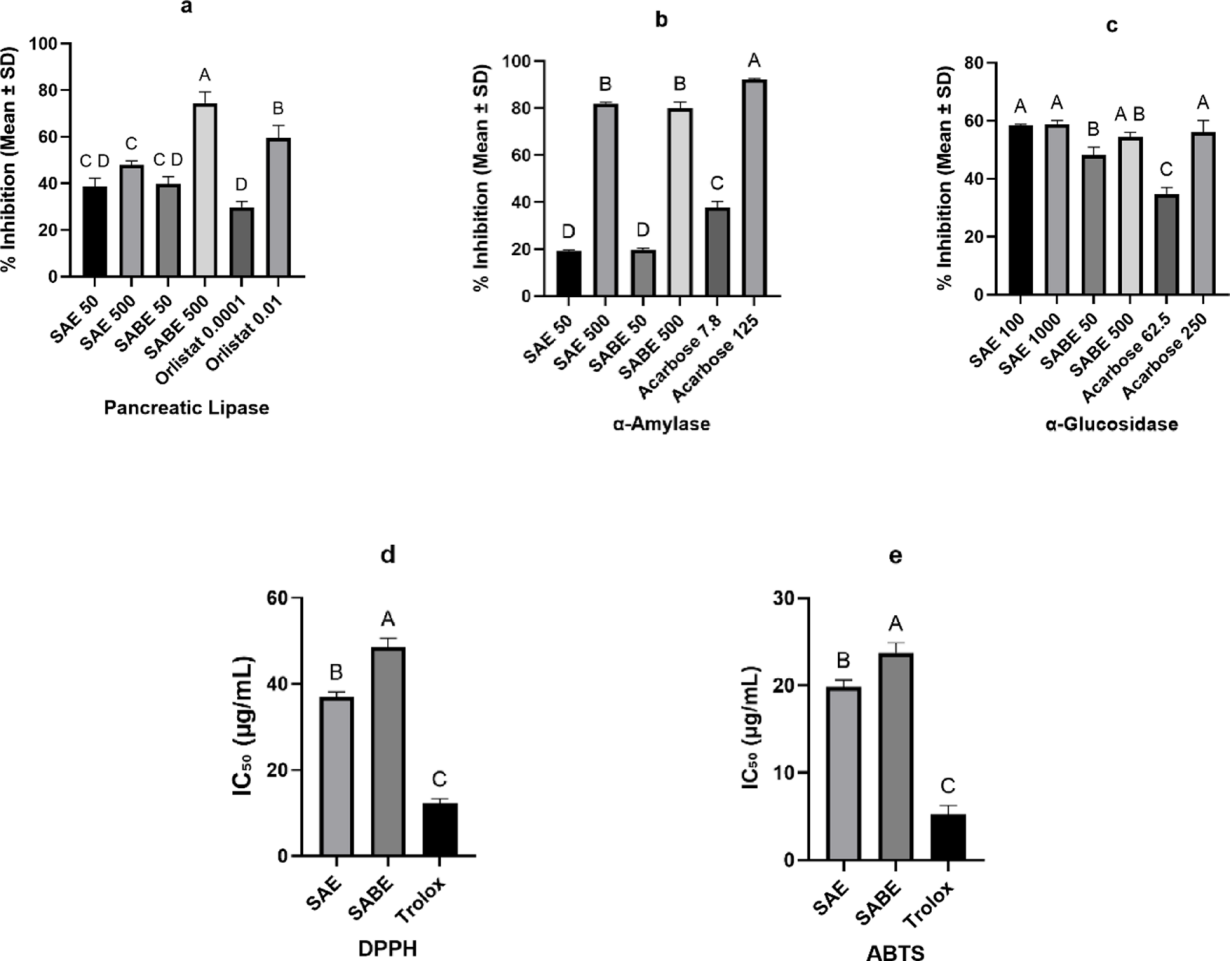
Tukey’s post HSD test for (**a**) pancreatic lipase, (**b**) α-amylase, (**c**) α-glucosidase, (**d**) DPPH, and (**e**) ABTS.

The antioxidant (DPPH and ABTS) and antihyperglycemic (α-amylase, α-glucosidase, and pancreatic lipase) assays results were reported as mean ± SD based on triplicate measurements (*n* = 3). Statistical significance of the scavenging and inhibitory activities was assessed using one-way analysis of variance (ANOVA) across all concentrations and treatment groups followed by Tukey’s Honest Significant Difference (HSD) post-hoc test for multiple comparisons using GraphPad Prism V.11.0.0.The ANOVA results revealed highly significant differences among all treatment groups (*p* < 0.0001), with F-values substantially exceeding the corresponding critical F-value, indicating strong evidence against the null hypothesis Table (S 1,2). When a significant F-test result was obtained and (*p* < 0.05), Tukey’s HSD post-hoc test was applied for multiple pairwise comparisons. This analysis enabled the detection of dose-dependent effects and facilitated comparison of SAE and SABE with reference standards (Trolox, Acarbose, and Orlistat). Differences were considered statistically significant at *p* < 0.05 and are denoted in the figures by letters (e.g., A, B, C); groups sharing the same letter are not significantly different at the 95% confidence level shown in (Fig. 8) and Table (S 3,4).

The statistical analysis, based on one-way ANOVA and Tukey’s HSD test (*p* < 0.05), showed that both SAE and SABE have a strong multitarget biological activity. For enzyme inhibition, SABE (500 *µ*g/mL) showed the highest lipase inhibition (74.49 ± 4.80%, Group A), significantly higher than orlistat (Group B). SAE showed a strong α-glucosidase inhibition, matching acarbose (Group A) at both tested concentrations. In the α-amylase assay, acarbose was the most effective (Group A), while SAE & SABE at 500 *µ*g/mL showed similar moderate activity (Group B). For antioxidant activity (DPPH and ABTS), Trolox (Group A) was the most effective, followed by SAE (Group B) and then SABE (Group C). SABE was more effective in lipase inhibition, while SAE demonstrated strong α-glucosidase inhibition along with superior antioxidant activity.

### Molecular docking study

A blind molecular docking strategy was implemented to explore the binding interactions between PL and sulfated flavonoids uniquely enriched in SABE. This approach enabled an in-depth characterization of the ligand‒protein complexes, with key parameters such as binding affinity (ΔG^a^), RMSD, interactions, interaction types, and their associated distances, as detailed in Table 3.

**Table 3:** Docked conformations of orlistat and sulfated flavonoids from SABE on the pancreatic lipase-colipase complex (PDB ID: 1LPB).

Compounds	ΔG^a^ (kcal/mol)	RMSD (Å)	Interactions	Type of Interaction	Distance (Å)/E (kcal/mol)
Isorhamnetin 3-*O*- sulfate	−12.47	1.36	His 263	H-donor	3.02/−7.0
Kaempferol 3-*O*-sulfate	−12.24	0.95	His 263	H-donor	3.14/−7.8
Mearnsetin 3-*O*-sulfate	−11.75	2.05	His 263	H-donor	3.18/−1.6
			Phe 77	pi-H	3.82/−0.9
Quercetin 3-*O*- sulfate	−11.60	2.32	His 263	H-donor	3.03/- 9.5
Orlistat	−11.54	1.5698	His 263	H-donor	3.27/−1.7
			Phe 77	H-donor	3.53/−0.5
			Arg 256	H-acceptor	3.47/−0.5

**Fig. 9:**
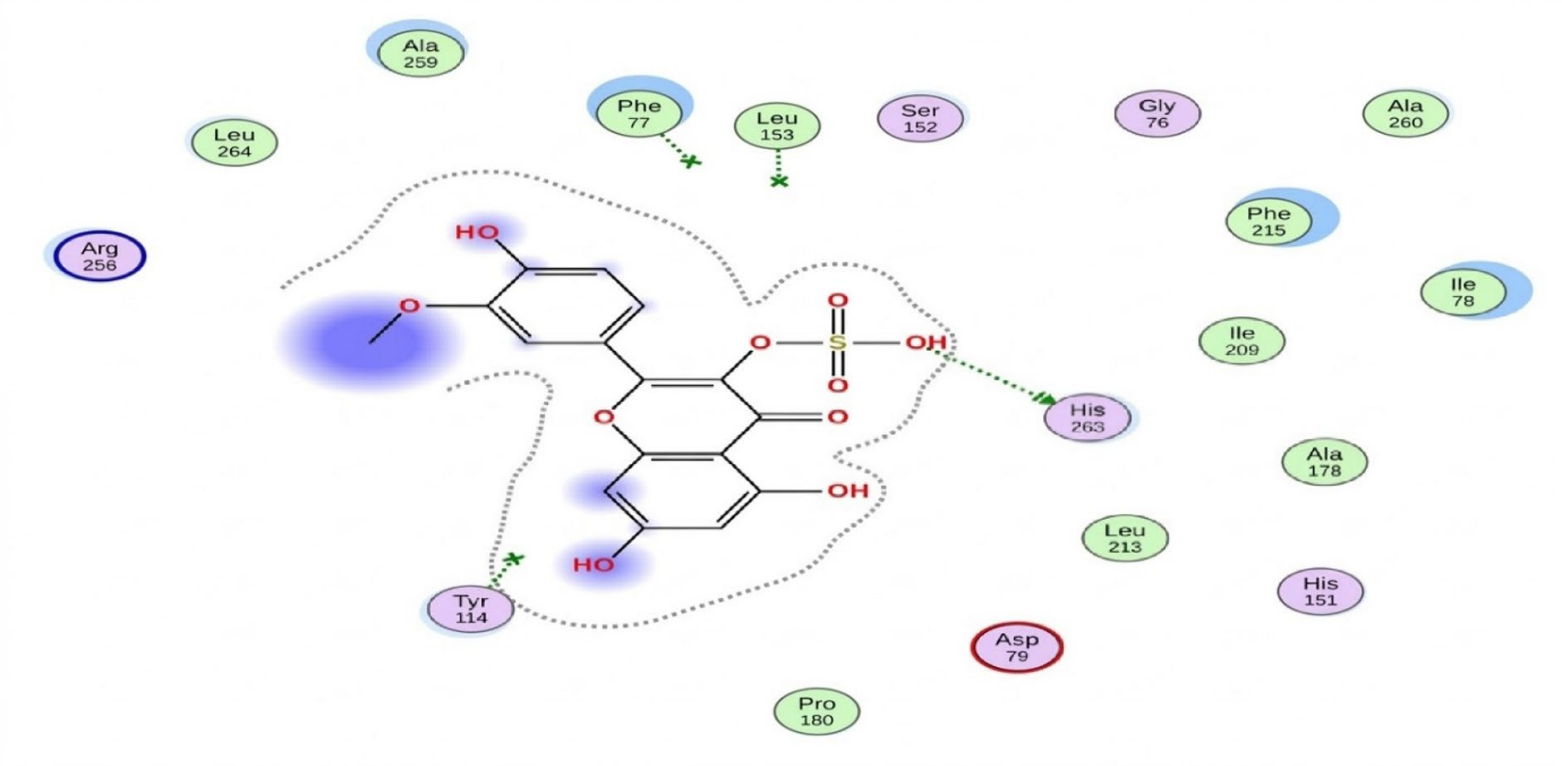
The 2D interactions of isorhamnetin-*O*-sulfate in the binding pocket of the pancreatic lipase-colipase complex (PDB ID: 1LPB).

Among the analysed compounds, isorhamnetin-3-*O*-sulfate **(**Fig. 9**)** exhibited the strongest binding affinity compared to orlistat (Fig. S15), with a ΔG^a^ of − 12.47 kcal/mol (fig. Its hydrogen bond interaction with His 263 (3.02 Å, − 7.0 kcal/mol) highlights a stable and favourable binding pose. The low RMSD value (1.36 Å) further supports its precise and stable binding conformation. Moreover, kaempferol 3-*O*-sulfate (Fig. S16) closely follows, with a ΔG^a^ of − 12.24 kcal/mol. It forms a strong hydrogen bond with His 263 (3.14 Å, − 7.8 kcal/mol) and achieves the lowest RMSD among all the compounds (0.95 Å), suggesting exceptional conformational stability.

## Conclusion

Microbial biotransformation of *S. australe* by *A. niger* induced pronounced remodelling of the metabolomic profile leading to the emergence of structurally diverse and discriminant metabolites. These biotransformation-driven chemical alterations were directly associated with altered antioxidant capacity and improved inhibitory activity against the lipase enzyme. Collectively, these findings underscore the potential of microbial biotransformation as a powerful and sustainable strategy for tailoring phytochemical composition and increasing specific biological efficacy. The catalytic versatility of *A. niger* highlights its value as a biotechnological platform for generating novel or enriched bioactive metabolites with therapeutic relevance to metabolic and lipid-related disorders. However, some limitations should be considered, as metabolite identification was based mainly on tentative LC-MS/MS annotation without full structural confirmation, and the biological evaluation was limited to in vitro assays and in silico docking. Moreover, the use of a single fungal strain under one fermentation condition may not capture the full spectrum of possible biotransformation outcomes. Future investigations should focus on targeted isolation and structural characterization of the key metabolites, integration of quantitative metabolomics to validate biomarkers of activity, and in vivo as well as mechanistic studies to fully elucidate their pharmacological potential and biosynthetic pathways.

## Supplementary Information

Below is the link to the electronic supplementary material.

Supplementary Material 1

## Data Availability

The datasets generated and/or analyzed in this study are available from the corresponding author upon reasonable request.

## Abbreviations

*A. niger*: *Aspergillus niger*
ABTS: 2,2’-Azino-bis (3-ethylbenzothiazoline-6-sulfonic acid)
BPC: Base peak chromatogram
Da: Dalton
DMSO: Dimethyl sulfoxide
DPPH: 2,2-diphenyl-1-picrylhydrazyl
GNPS: Global Natural Products Social Molecular Networking
HCA: Hierarchical clustering analysis
MN: Molecular network
PBS: Phosphate-buffered saline
PCA: Principal component analysis
PLS-DA: Partial least squares–discriminant analysis
OPLS-DA: Orthogonal partial least squares–discriminant analysis
R_t_: Retention time
*S*. *australe*: *Syzygium australe*
SABE: *Syzygium australe* biotransformed extract
SAE: *Syzygium australe* leaves extract
UPLC/T-TOF-MS/MS: Ultra-Performance Liquid Chromatography Triple Time-of-Flight - Mass Spectrometry
