# Supplementary material for: Microbial biotransformation of Syzygium australe modifies metabolomic profile assessed with multivariate analysis and molecular networking: In vitro and computational studies
Source: Sci Rep. 2026 Jun 14;16:18372. doi: 10.1038/s41598-026-55423-x (PMC13265851; doi:10.1038/s41598-026-55423-x)
Supplement: Supplementary file 1 — Supplementary Material 1 [file 41598_2026_55423_MOESM1_ESM.docx]

**
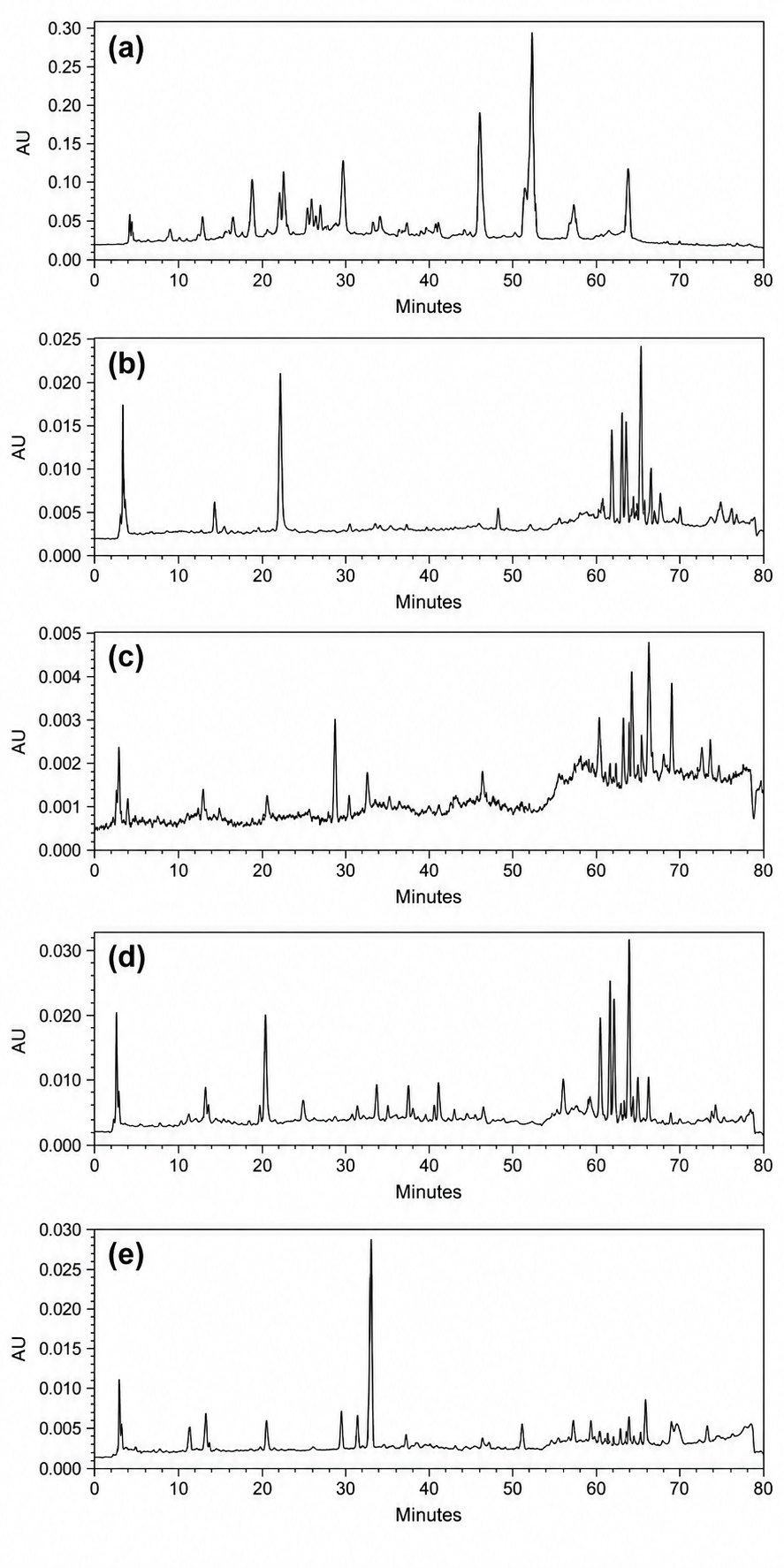
**

**Fig. (S1):** HPLC chromatograms of (a); substrate extract at 0 days, (b); A. niger control in SDB without substrate, (c–e); substrate extract incubated with A. niger for 14 days (c), 21 days (d), and 28 days (e).

**
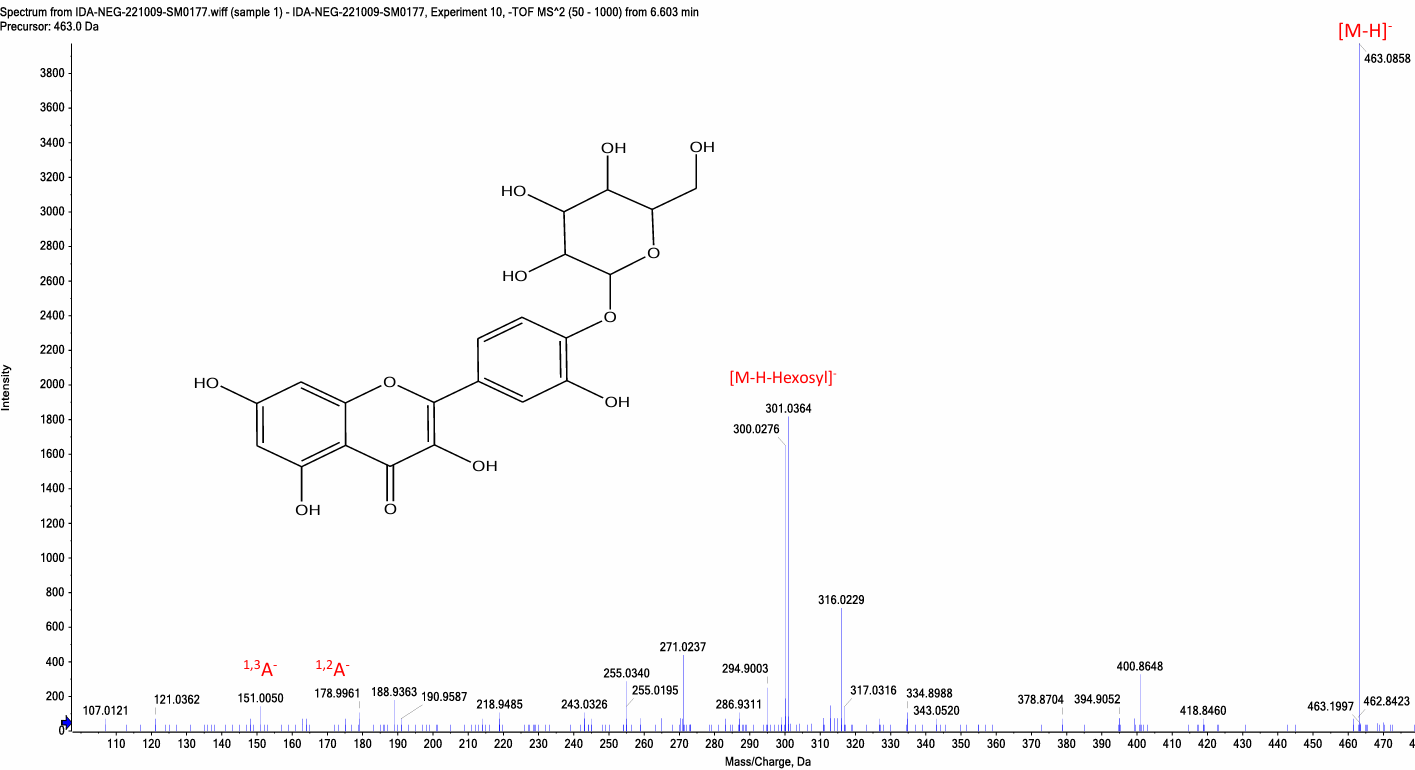
**

**Fig. (S2):** MS/MS spectra of quercetin-*O*-hexoside


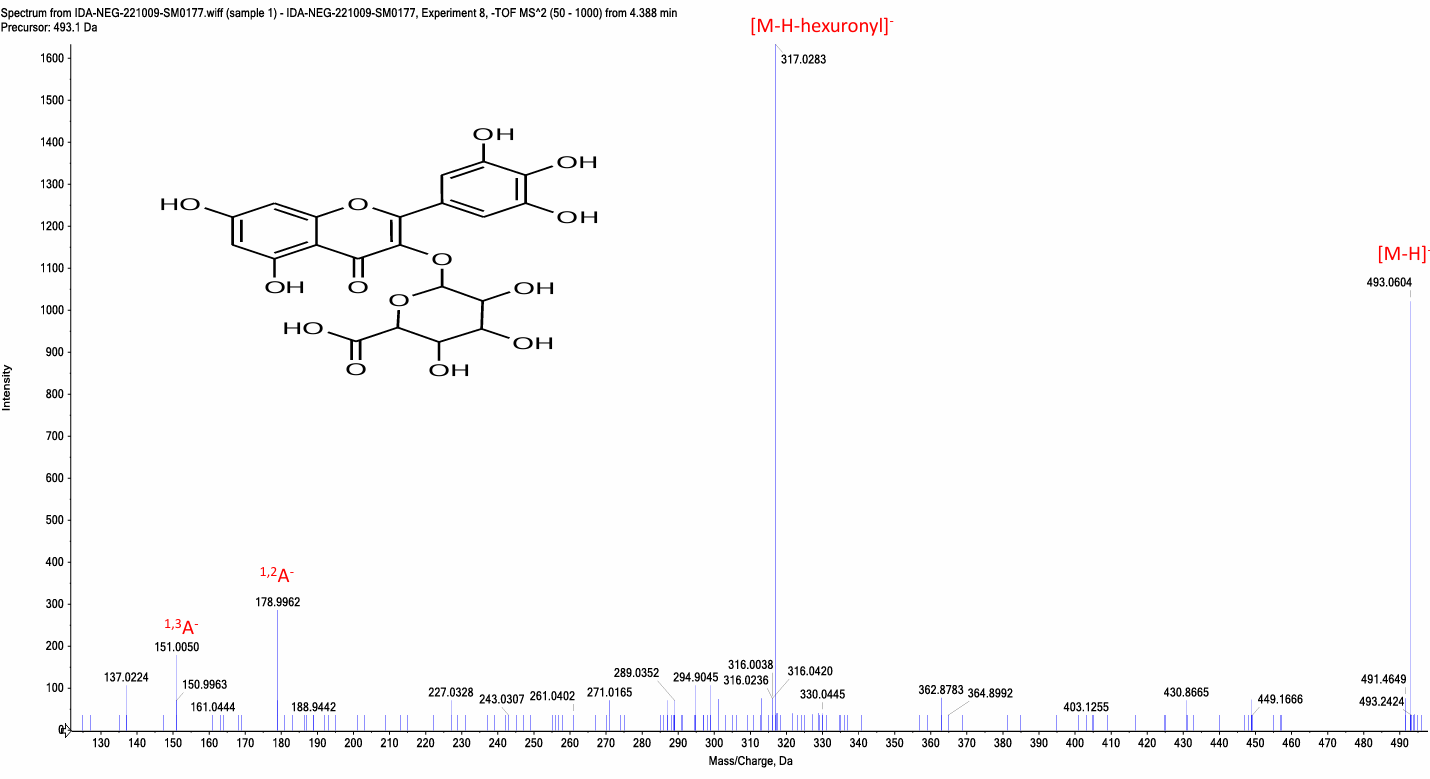
 **Fig. (S3):**MS/MS spectra of myricetin -*O*-hexuronide

**
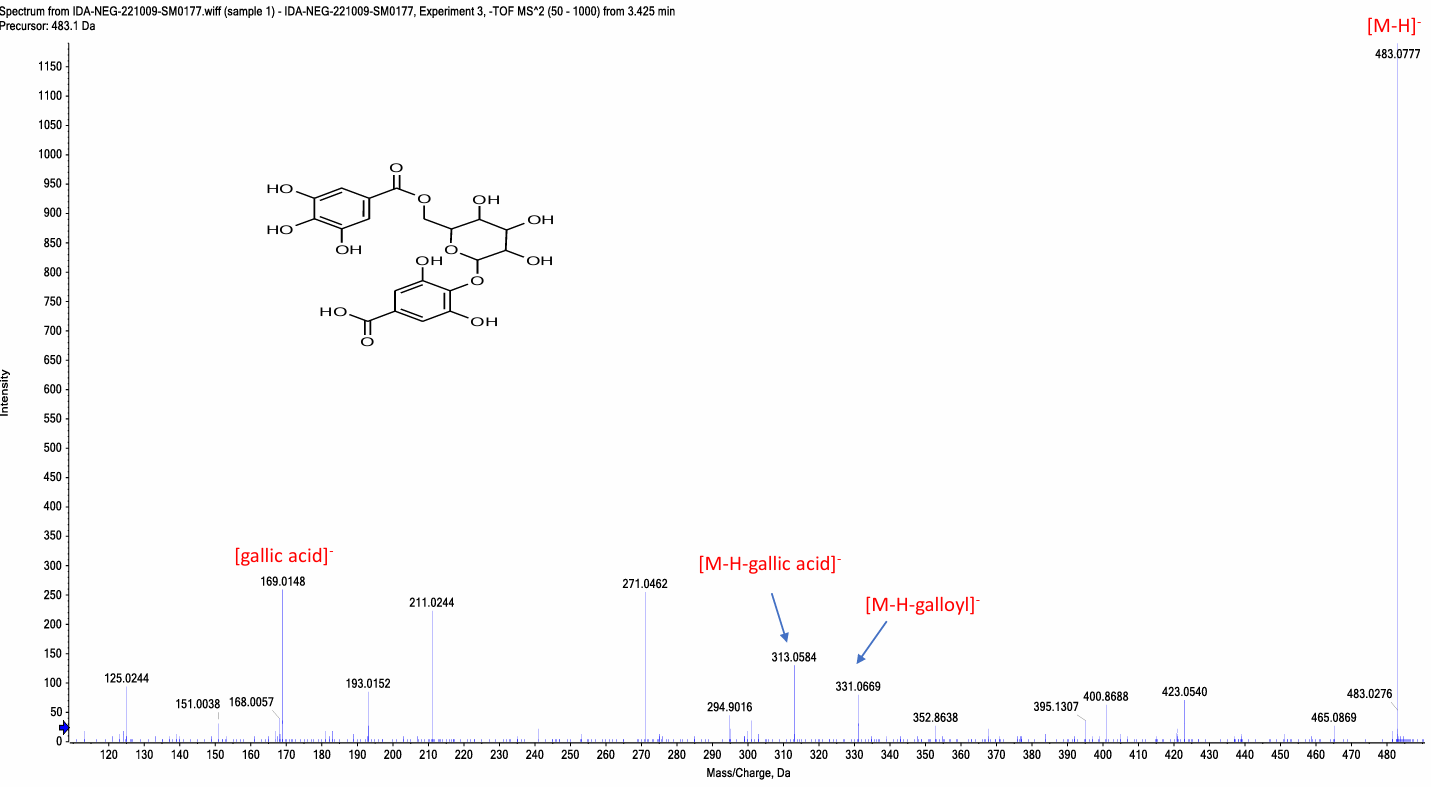
**

**Fig. (S4):**MS/MS spectra of gallic acid-*O*-(6 galloyl hexoside)

**
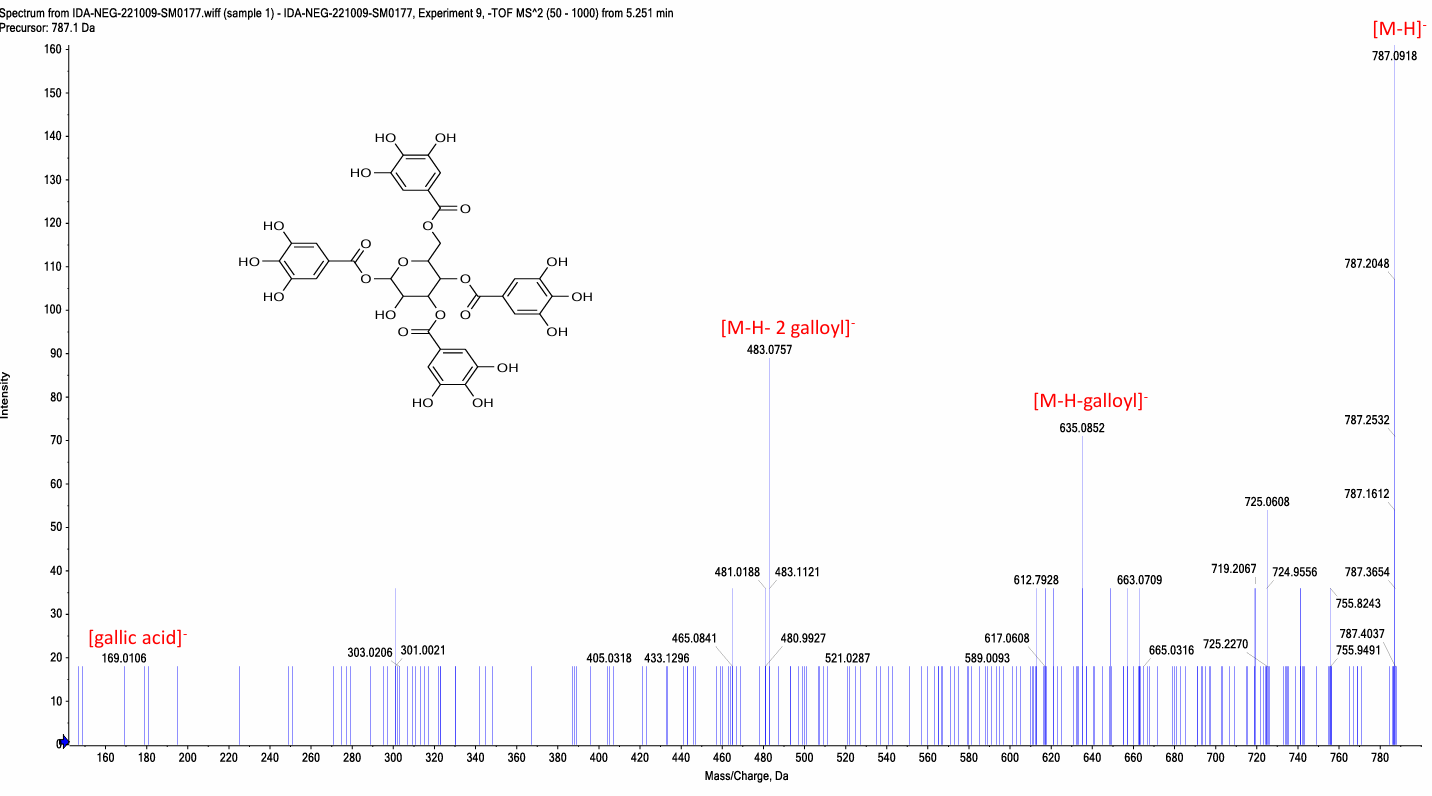
**

**Fig. (S5):**MS/MS spectra of tetra-*O* -galloyl -hexoside

**
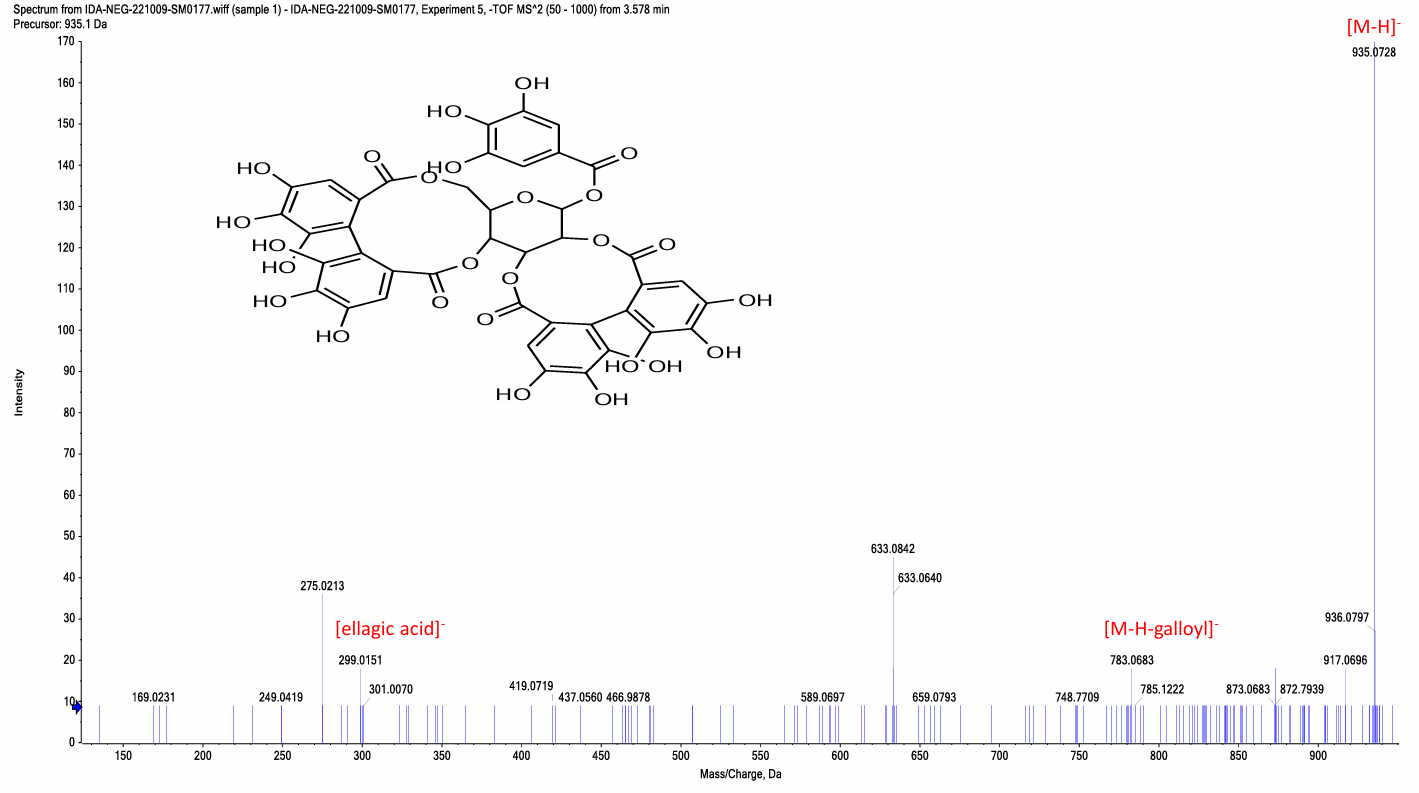
**

**Fig. (S6):** MS/MS spectra of casuarictin

**
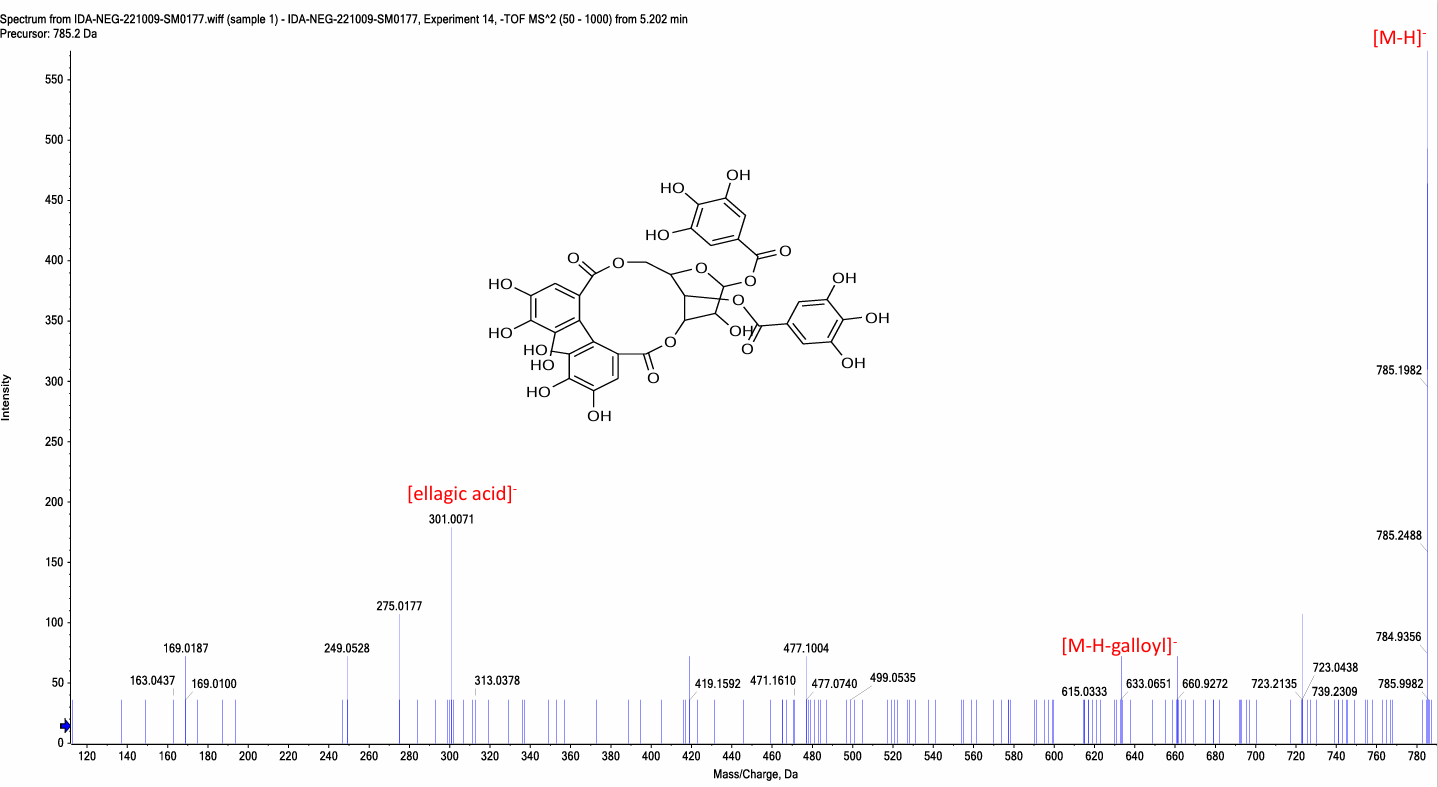
**

**Fig. (S7):**MS/MS spectra of di-galloyl-hexahydroxydiphenoyl-hexoside


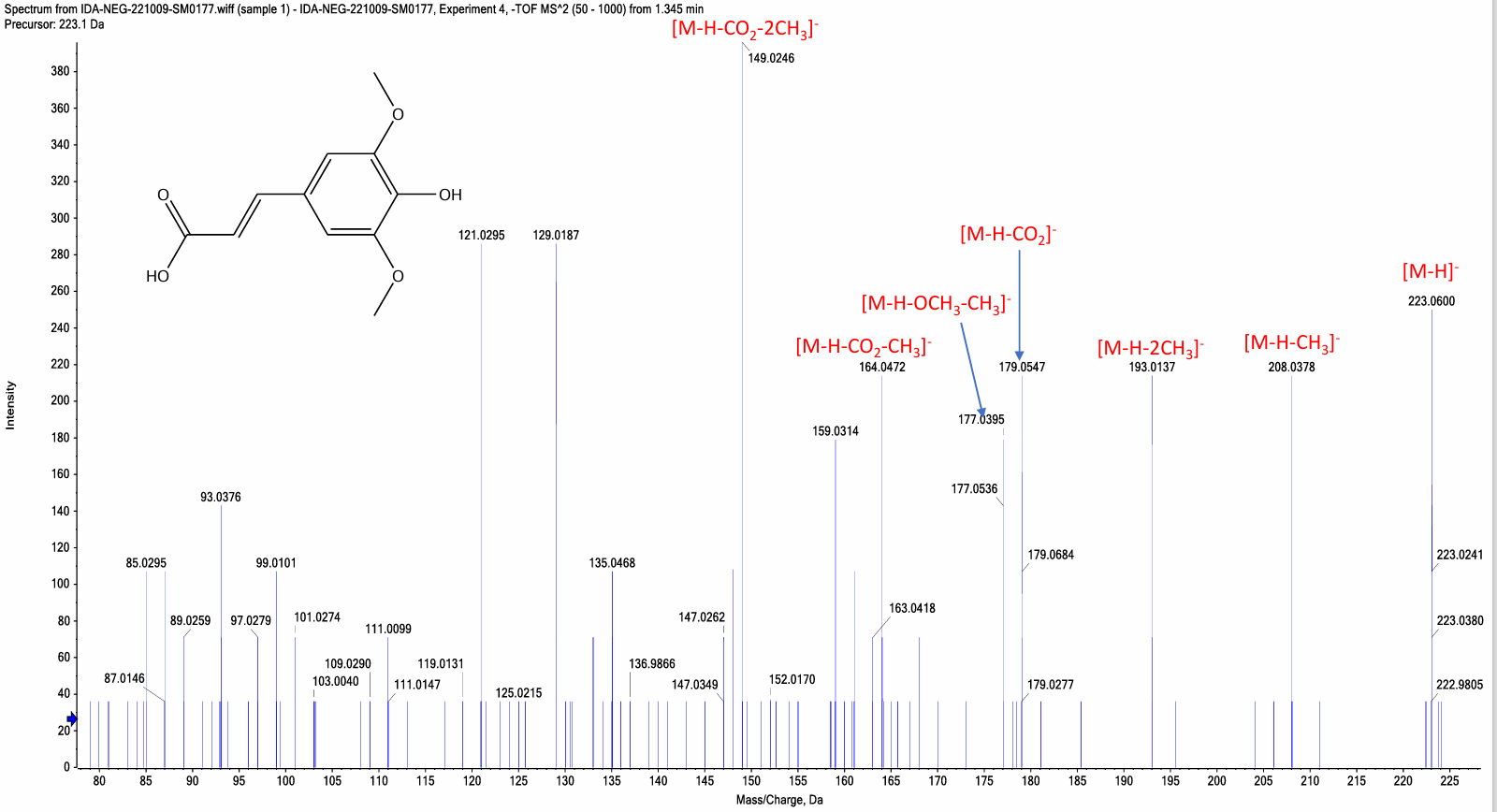


**Fig. (S8):**MS/MS sinapic acid


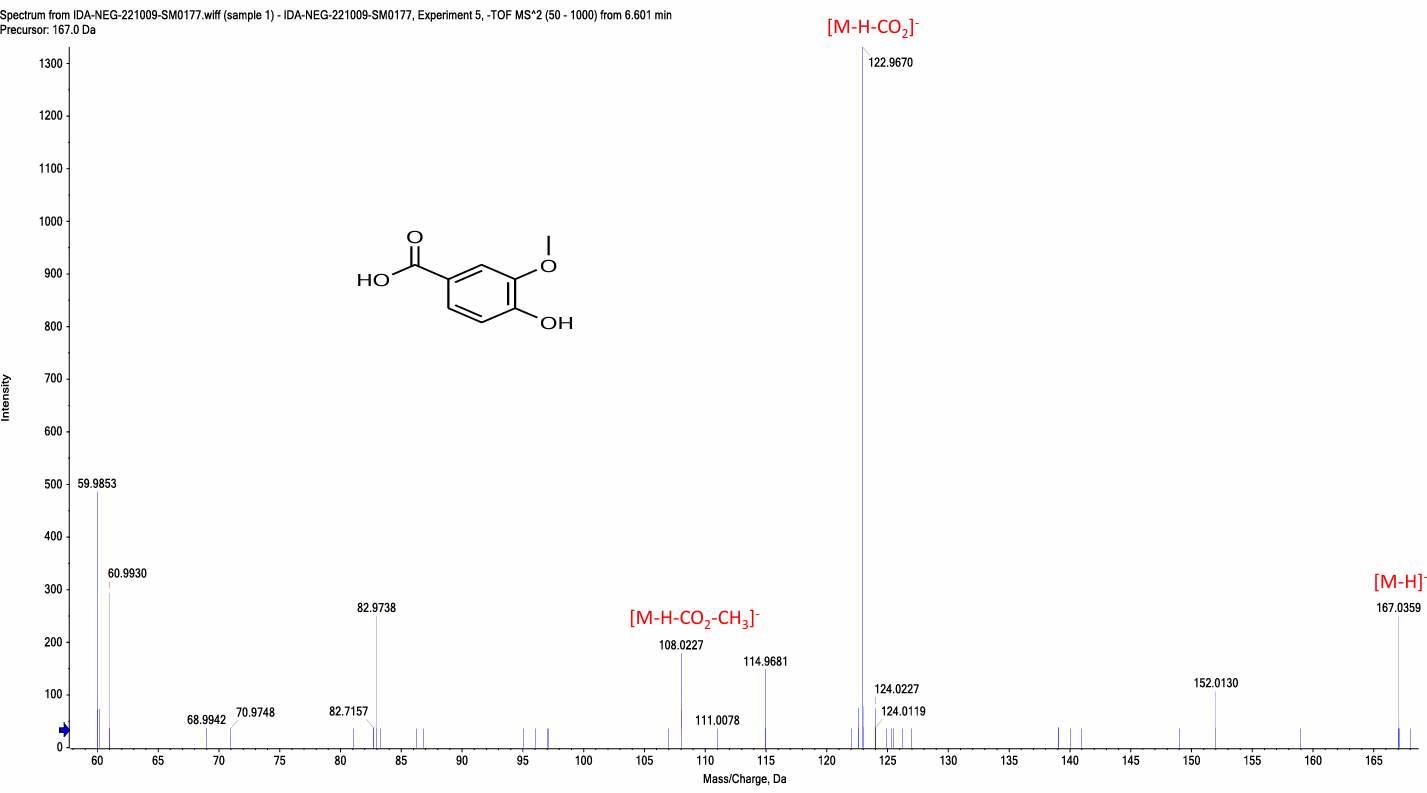


**Fig.** (S9):MS/MS spectra of vanillic acid


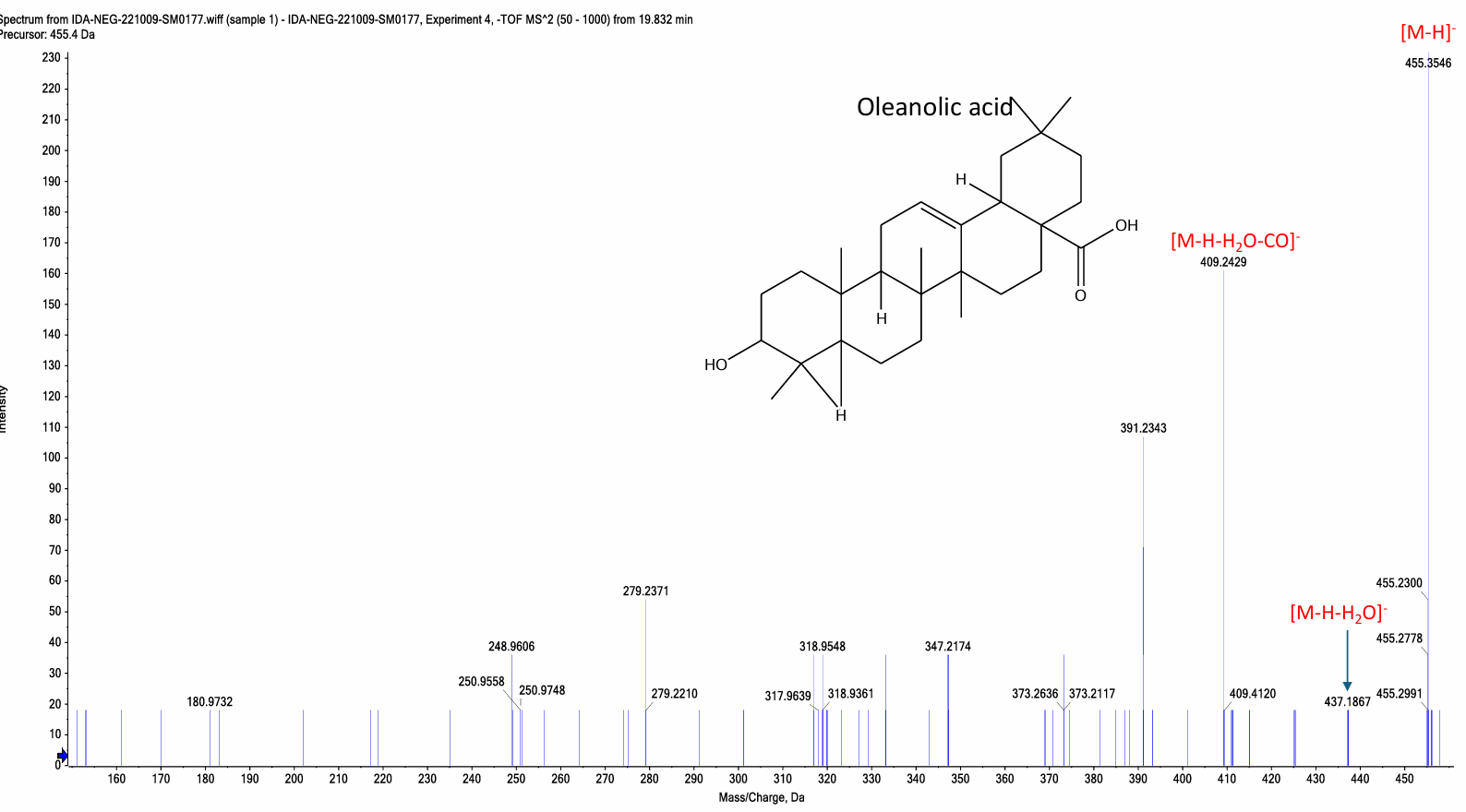


**Fig. (S10):** MS/MS spectra of oleanolic acid

**
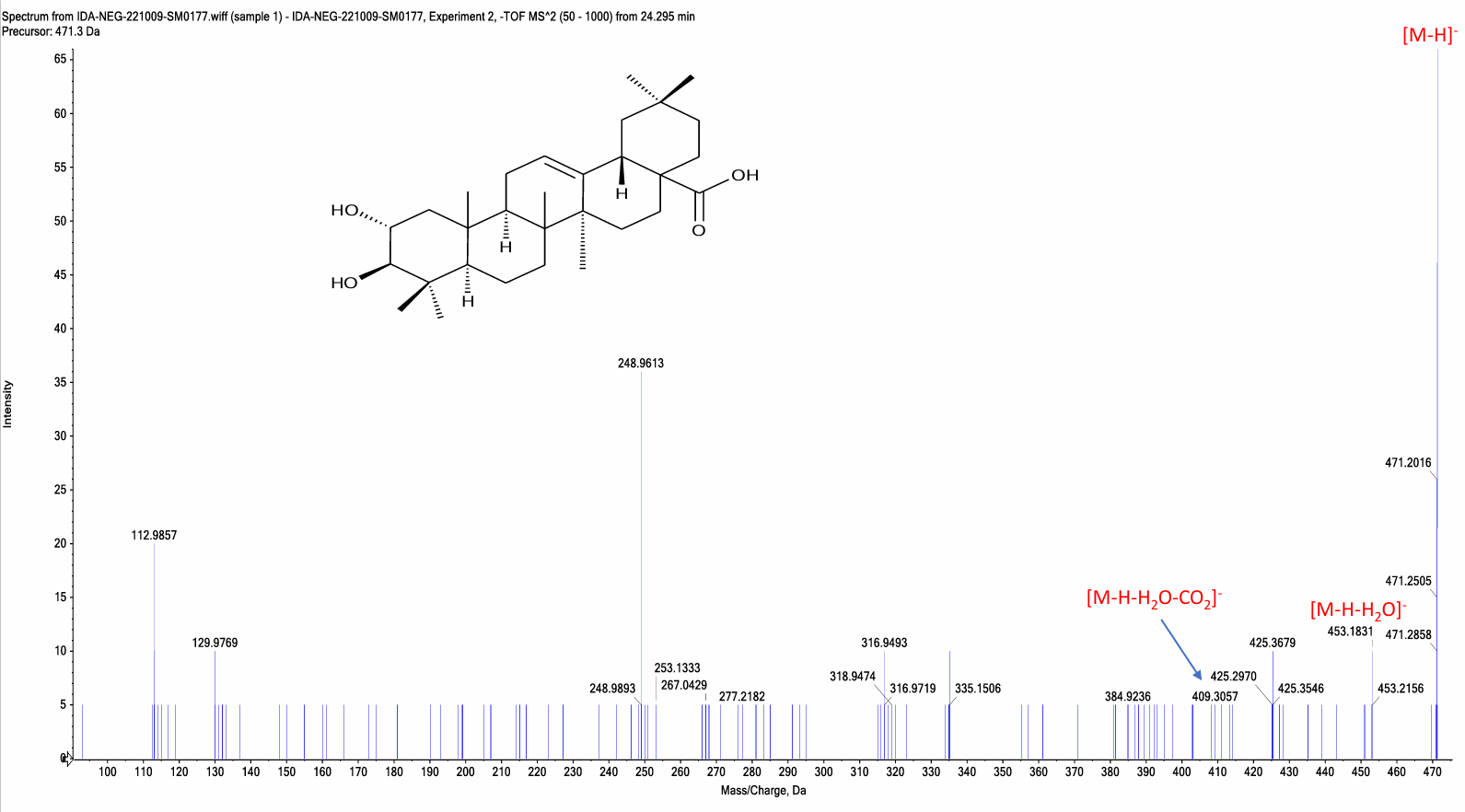
**

**Fig. (S11):**MS/MS spectra of maslinic acid


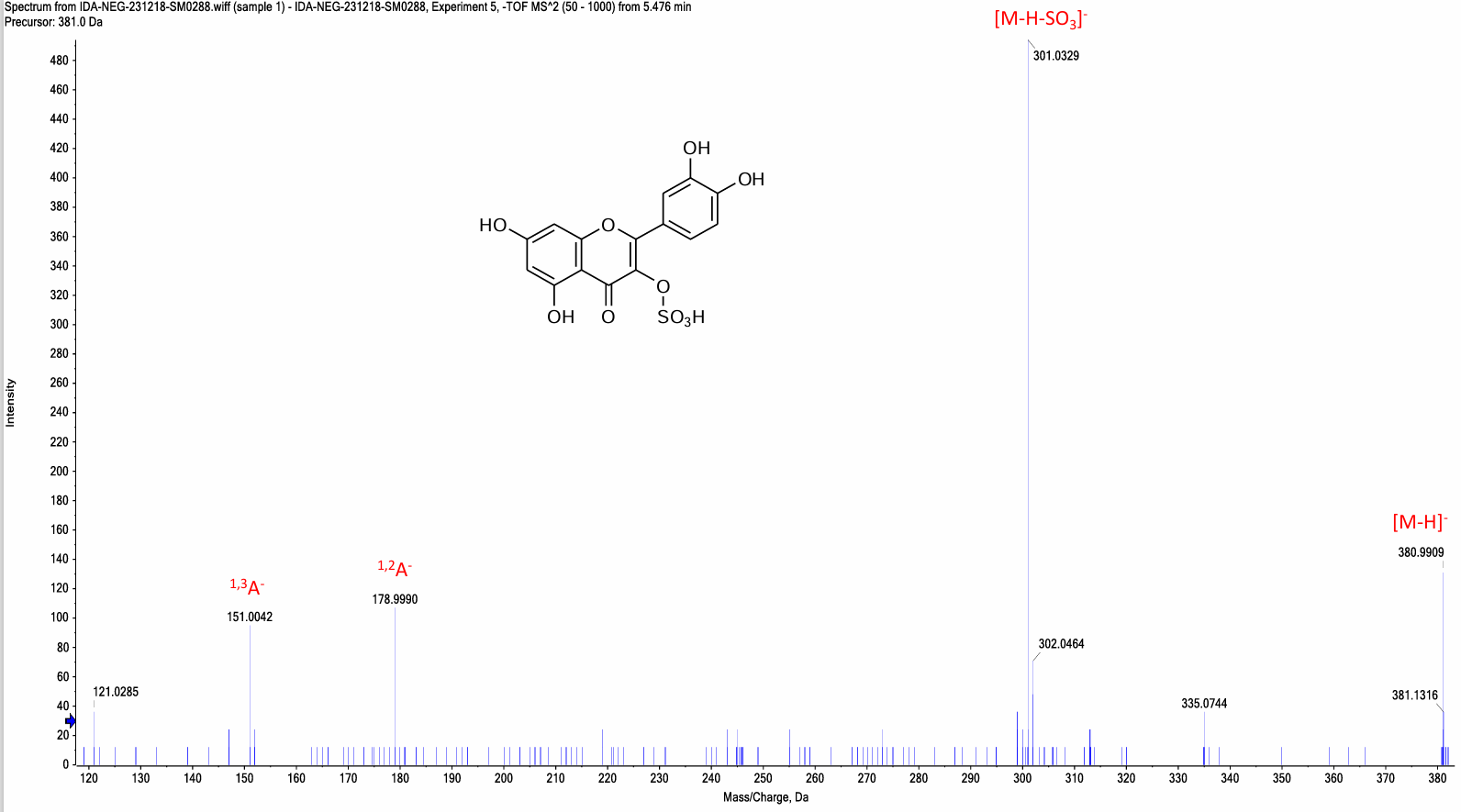


**Fig. (S12):** MS / MS spectra of quercetin *O*-sulfate


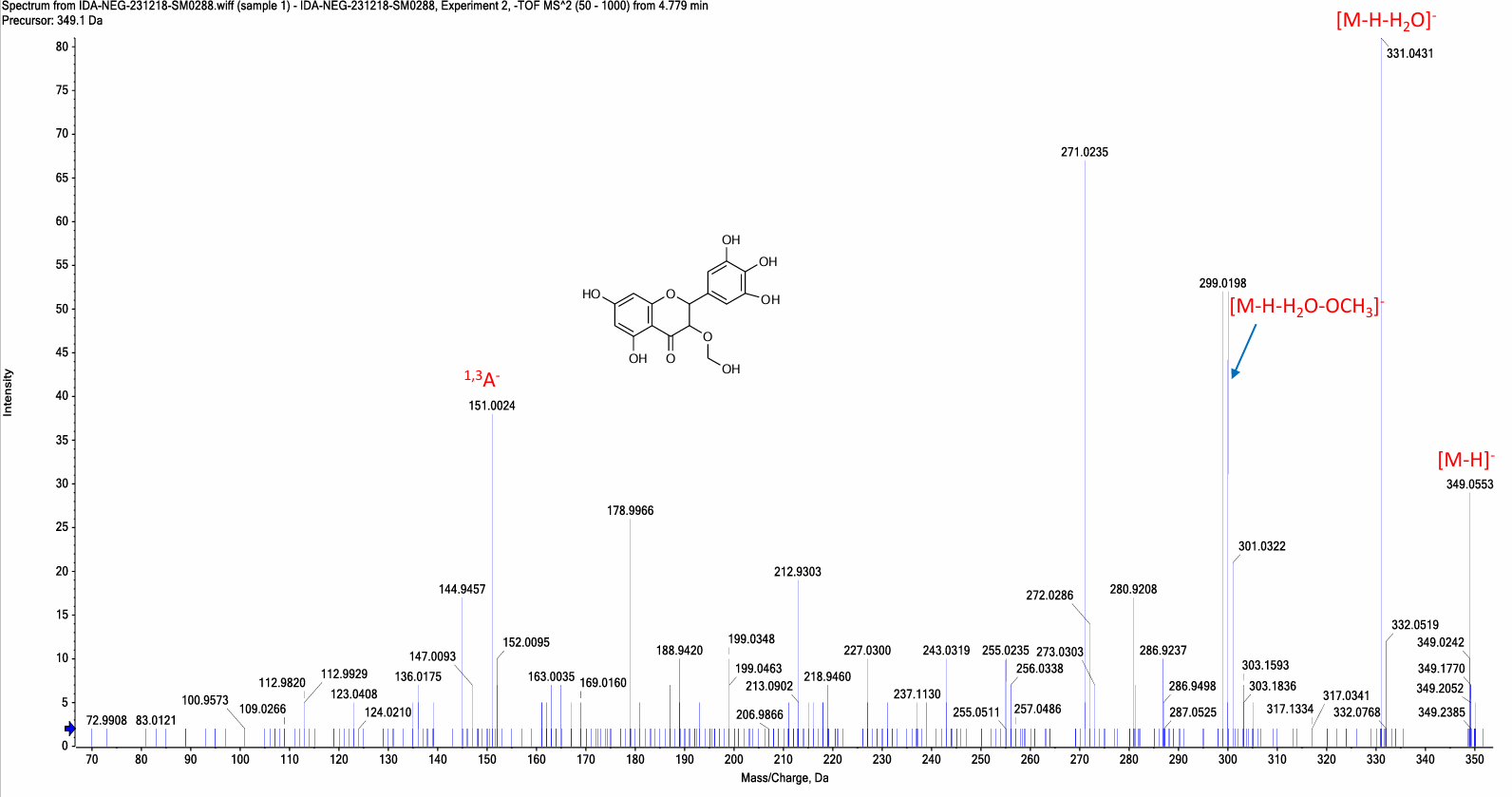
 **Fig. (S13):** MS /MS spectra of Pentahydroxy-3-hydroxymethoxy-flavanone

**Fig (S14) (a)** Overview of the OPLS-DA model showing model performance metrics (b) Permutation Test for OPLS-DA Model Validation


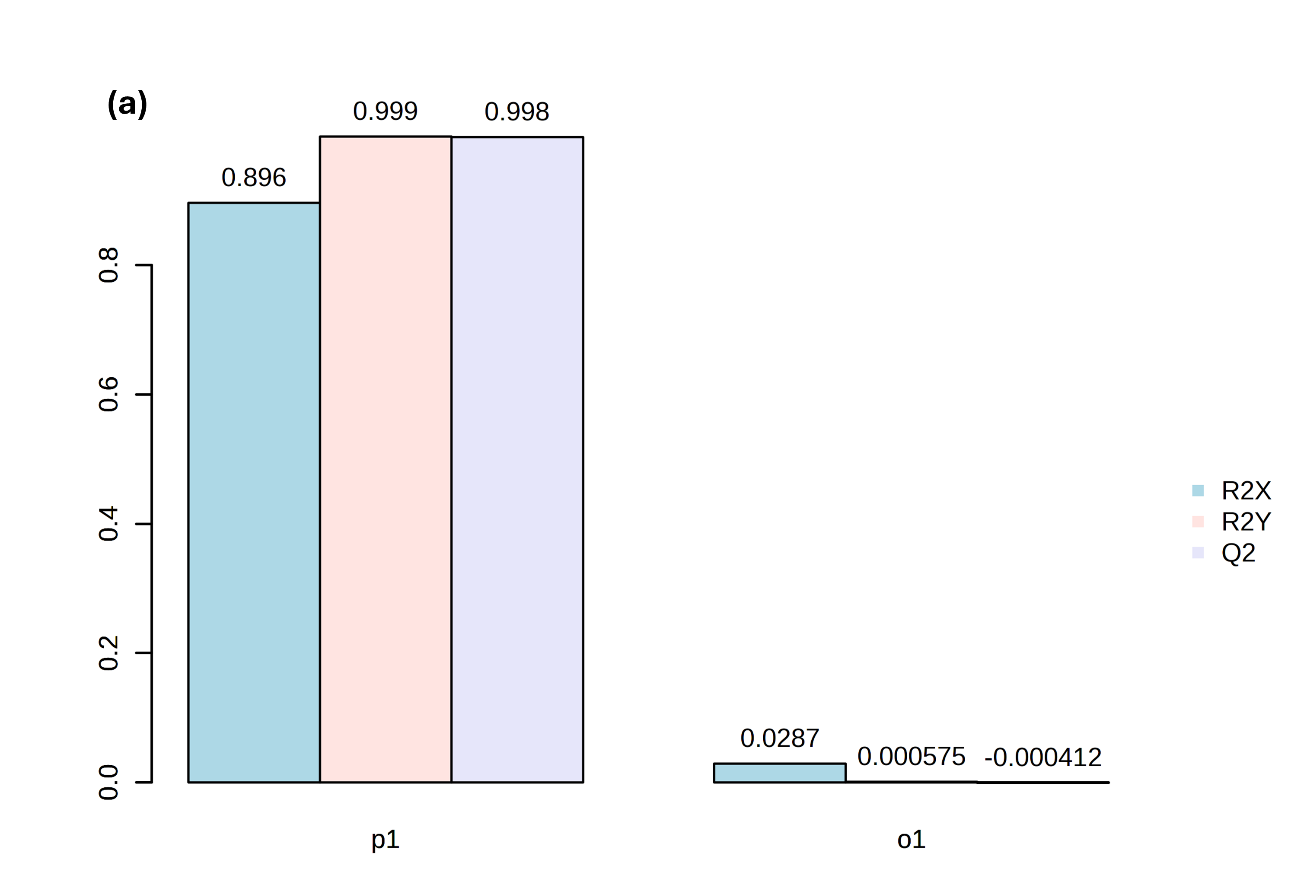

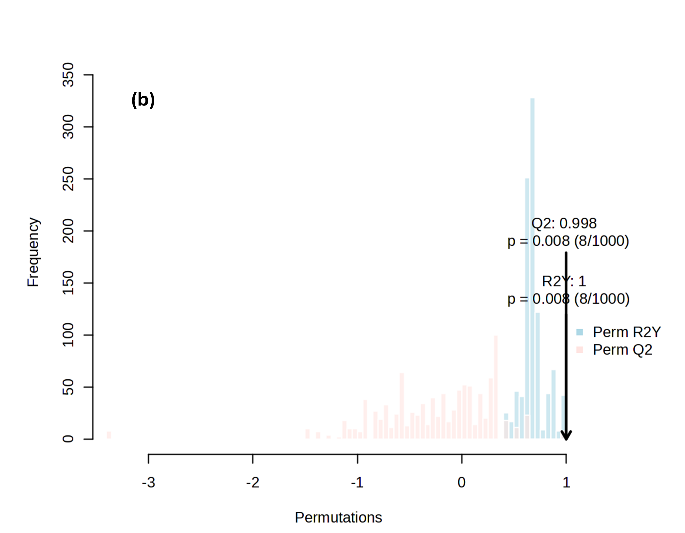


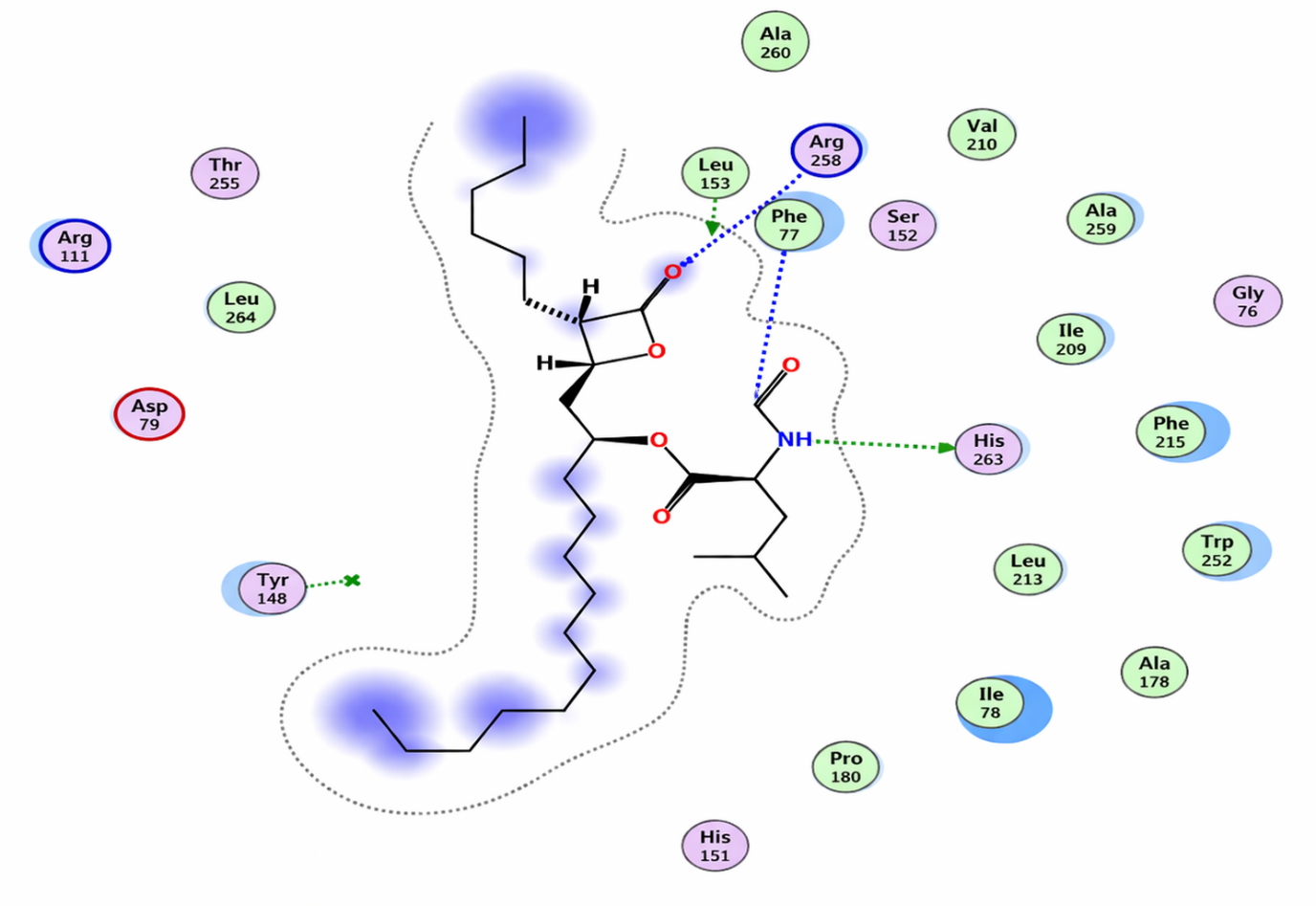


**Fig. (S15):** The 2D interactions of orlistat in the binding pocket on pancreatic lipase-colipase complex (PDB ID: 1LPB).


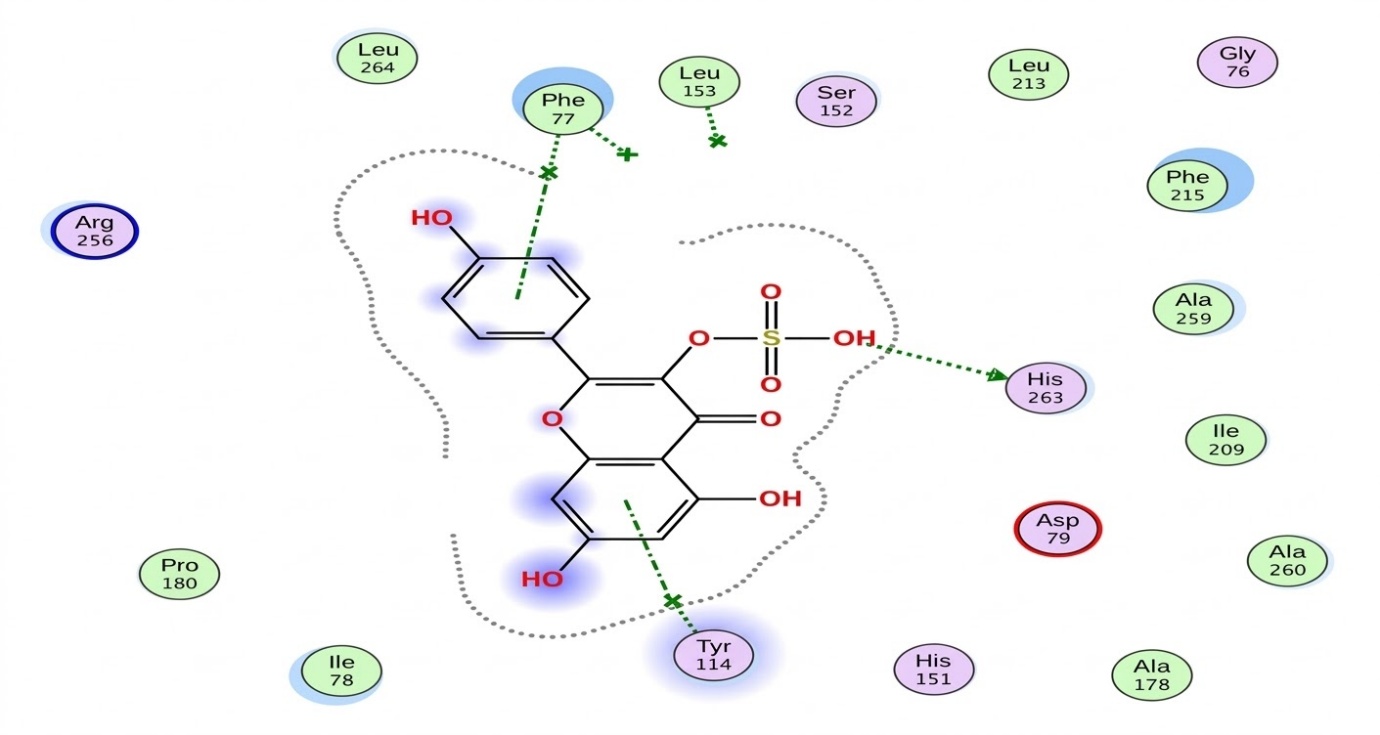


**Fig. (S16):** The 2D interactions of kaempferol-*O*-sulfate in the binding pocket.on pancreatic lipase-colipase complex (PDB ID: 1LPB).

Supplementary Tables

**Table S1**: One-way ANOVA results for lipase, α-amylase, and α-glucosidase inhibition by SAE, SABE, Orlistat, and Acarbose.

| **Enzyme** | **df ^a^** | **SS ^b^** | **MS ^c^** | **F** | **P** |
| --- | --- | --- | --- | --- | --- |
| **Lipase Inhibition** | | | | | |
| Between Groups | 5 | 3,961.24 | 792.25 | 56.19 | <0.0001 |
| Within Groups | 12 | 169.19 | 14.10 | — | — |
| **Total** | 17 | 4,130.43 | — | — | — |
| **α-Amylase Inhibition** | | | | | |
| Between Groups | 5 | 16,668.12 | 3,333.62 | 1,370.17 | <0.0001 |
| Within Groups | 12 | 29.20 | 2.43 | — | — |
| **Total** | 17 | 16,697.32 | — | — | — |
| **α-Glucosidase Inhibition** | | | | | |
| Between Groups | 5 | 1,277.81 | 255.56 | 47.07 | <0.0001 |
| Within Groups | 12 | 65.15 | 5.43 | — | — |
| **Total** | 17 | 1,342.96 | — | — | — |

**^a^** Degrees of Freedom

**^b^** Sum of Squares

**^c^** Mean Square

**Table S2.** One-way ANOVA results for DPPH and ABTS inhibition by SAE, SABE, and Trolox.

| **Assay** | **Source of Variation** | **df ^a^** | **SS ^b^** | **MS ^c^** | **F** | **p-value** |
| --- | --- | --- | --- | --- | --- | --- |
| **DPPH** | Between Groups | 2 | 2049 | 1025 | 447.4 | < 0.0001 |
|  | Within Groups | 6 | 13.74 | 2.92 | — | — |
|  | Total | 8 | 2063 | — | — | — |
| **ABTS** | Between Groups | 2 | 572.0 | 286.0 | 286.6 | < 0.0001 |
|  | Within Groups | 6 | 5.987 | 0.9978 | — | — |
|  | Total | 8 | 578.0 | — | — | — |

**^a^** Degrees of Freedom

**^b^** Sum of Squares

**^c^** Mean Square

**Table S3**: Tukey’s HSD grouping (P < 0.05) of % inhibition (mean ± SD) for SAE, SABE, and standard drugs (Orlistat and Acarbose) against pancreatic lipase, α-amylase, and α-glucosidase at different concentrations

| **Enzyme Assay** | **Sample Treatment** | **Concentration (µg/mL)** | **% Inhibition** **(Mean ± SD)** | **Tukey Grouping** |
| --- | --- | --- | --- | --- |
| **Pancreatic Lipase** | SABE | 500 | 74.49 ± 4.80 | A |
|  | Orlistat | 0.01 | 59.48 ± 5.41 | B |
|  | SAE | 500 | 47.98 ± 1.73 | C |
|  | SABE | 50 | 39.77 ± 3.13 | CD |
|  | SAE | 50 | 38.66 ± 3.66 | CD |
|  | Orlistat | 0.0001 | 29.76 ± 2.47 | D |
| **α-Amylase** | Acarbose | 125 | 92.29 ± 0.32 | A |
|  | SAE | 500 | 81.95 ± 0.59 | B |
|  | SABE | 500 | 79.93 ± 2.69 | B |
|  | Acarbose | 7.8 | 37.80 ± 2.48 | C |
|  | SABE | 50 | 19.69 ± 0.77 | D |
|  | SAE | 50 | 19.29 ± 0.41 | D |
| **α-Glucosidase** | SAE | 1000 | 58.84 ± 1.28 | A |
|  | SAE | 100 | 58.47 ± 0.38 | A |
|  | Acarbose | 250 | 56.13 ± 3.94 | A |
|  | SABE | 1000 | 54.41 ± 1.64 | AB |
|  | SABE | 100 | 48.21 ± 2.70 | B |
|  | Acarbose | 62.5 | 34.67 ± 2.30 | C |

**Table S4**: Tukey’s HSD grouping (p < 0.05) for the DPPH and ABTS IC₅₀ values of SAE, SABE, and Trolox.

| **Sample** | **DPPH IC₅₀ (µg/mL)**  **(Mean ± SD)** | **Tukey Group** | **ABTS** **IC₅₀ (µg/mL)**  **(Mean ± SD)** | **Tukey Group** |
| --- | --- | --- | --- | --- |
| Trolox | 12.32 ± 1.01 | A | 5.24 ± 1.03 | A |
| SAE | 36.96 ± 1.20 | B | 19.80 ± 0.85 | B |
| SABE | 48.50 ± 2.10 | C | 23.79 ± 1.10 | C |
